# Social prescribing for refugee populations: a rapid realist review of international evidence

**DOI:** 10.3389/fpubh.2026.1758335

**Published:** 2026-05-08

**Authors:** Victoria Touzel, Doreen Reifegerste, Luisa Bartz, Anna-Lena Esser, Kerryn Husk

**Affiliations:** 1 AG4 Prevention and Health Promotion, Universität Bielefeld, Bielefeld, Germany; 2 Faculty of Health, University of Plymouth, Plymouth, United Kingdom

**Keywords:** asylum seeker, realist review, refugee, social capital, social prescribing

## Abstract

**Background:**

Social prescribing offers potential for addressing social determinants of health and supporting health equity among disadvantaged groups. However, evidence for refugee populations remains limited, despite this group facing profound social and systemic barriers. This rapid realist review synthesizes social prescribing and comparable social-capital based intervention evidence to address this gap.

**Methods:**

We conducted a RAMESES-compliant rapid realist review supported by an expert advisory board. Searches across six databases (2014–2024) were supplemented by grey literature and citation chasing strategies. Eligible studies included refugee, asylum-seekers and forcibly displaced populations engaged in either formal social prescribing or comparably operationalized social-capital interventions. Synthesis developed Context-Mechanism-Outcome configurations organized into intervention families and articulated as If-Then programme theories.

**Results:**

From 7,436 records, 39 studies contributed to synthesis. As anticipated, formal social prescribing evidence was limited; findings are therefore substantially theory-informed extrapolations from social-capital interventions rather than direct evidence. Five intervention families were identified, spanning barrier-reduction, co-produced navigation, trauma-responsive, community-connected, and skills-training approaches. Of 15 prioritized programme theories, five demonstrated strong-to-moderate evidence, with the majority of actionable insights concentrated in appointment and onward referral stages.

**Discussion:**

Social prescribing appropriateness and effectiveness depends on alignment between contextual barriers, activated mechanisms, and support infrastructure. Three key cross-cutting concepts were identified: co-production as bidirectional exchange; safety as spatial and relational; and enabler provision as a barrier-matching requirement. Evidence limitations restrict wider generalizability, particularly regarding population reporting, further marginalized subgroups, and context considerations.

**Conclusion:**

Social prescribing for refugee support requires distinct consideration through adapted design, targeted barrier reduction, workforce investment, and genuine co-production with refugee-serving communities, rather than transferring dominant-population models. Evidence is strongest for in-appointment and onward referral strategies; access pathways into social prescribing remain the most critical evidence gap.

## Graphical abstract

**Figure:**
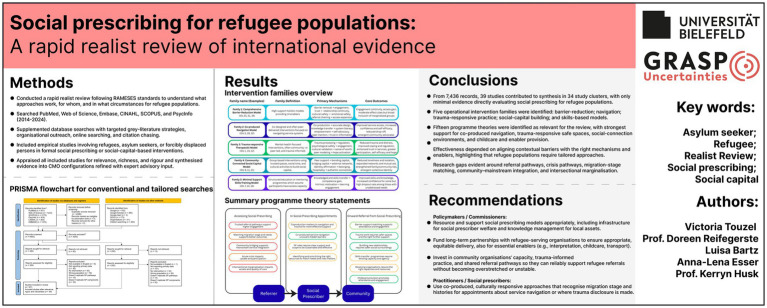

## Introduction

Social prescribing has been implemented at scale in multiple countries, with policy and financial traction in many others, as a health system component addressing the social determinants of health through non-clinical forms of support (1). By connecting individuals to activities such as arts, sports, peer groups, or advice services, social prescribing seeks to impact health and wellbeing, reduce strain on health services through reducing loneliness, strengthening social networks, and promoting holistic wellbeing (2, 3). Refugee populations have remained largely absent from formal evaluations (4), despite facing profound social and systemic barriers (5–8). This rapid realist review addresses this urgent gap by assessing what approaches to social prescribing work, for whom, and under what circumstances for refugee populations. Given the likely sparsity of SP-related evidence, this review also considers findings from social-capital based interventions operationalized in ways we consider comparable to social prescribing.

Two key concepts underpin this review, namely social prescribing and social capital. In this research, social prescribing refers to referral, typically from health settings into community-based, non-clinical activities or services designed to support health and wellbeing (9, 10). As such, it operates as a bridge between healthcare and community infrastructures, aiming both to empower individuals and to relieve pressure on clinical services (1, 11). In contrast, social capital is a more abstracted concept. It is here defined as the resources embedded in social networks, such as trust, reciprocity, and social support, which individuals can mobilize to improve wellbeing and participation (12, 13). Social capital is crucial to mitigating isolation, supporting agency, and fostering integration, and thereby clearly relevant for refugee populations (12, 14). Interventions that build social capital, such as peer support activities, may therefore provide insights directly relevant to social prescribing for these populations.

In this review, the term refugee is used in a broad and inclusive sense. We include people who have sought protection outside their country of origin, such as those formally recognized as refugees, individuals awaiting decisions on asylum applications, and others with precarious legal status, where these are often described as asylum seekers or forced migrants (15, 16). This choice of definition acknowledges the diversity of legal and lived realities, while recognizing shared challenges arising from displacement, lived experience of poverty, trauma, and resettlement contexts (8, 14, 17).

## Background

Research on social prescribing has expanded rapidly in recent years and evidence suggests that social prescribing can improve wellbeing, reduce loneliness, and strengthen social connections, while in some cases also contributing to reduced health service use (2, 18–22). However, the state of the art also highlights persistent challenges. These general limitations include uneven access to services (with notable data gaps), inconsistent referral practices, and limited clarity on which mechanisms generate benefits in different contexts or for different (marginalized) populations (23–26). Taken together, the literature portrays social prescribing as a promising but complex health system approach that depends heavily on local context, practitioner engagement, and the availability of skilled community resource and capacity (19, 27, 28).

From an equity perspective, these limitations are particularly concerning. Social prescribing has expanded across diverse health systems from the UK to Australia, Canada, the Netherlands and further; yet, the evidence base has been generated predominantly with majority populations in high-income settings, regardless of national context (1, 11). Across these contexts, marginalized populations who are known to face greater barriers to healthcare and community participation have seldom been the focus of evaluation. Refugee populations exemplify this paradox: they often experience legal precarity, hostile policy environments, poverty, and limited access to health and social services, but their needs have yet to be addressed by social prescribing literature. Where migrated populations have been addressed in research, evidence remains thin, non-specific, and under-analysed, leading to repeated calls for more evidence, including in the EU context (4, 24, 29, 30). Without explicit attention to refugee populations, non-tailored social prescribing approaches risk inadvertently exacerbating underlying inequities by continuing to primarily serve those who are already better positioned to engage with health systems and community infrastructures. Given this clear need for further research, this review therefore responds to this need for first exploratory evidence.

In parallel, evidence from social-capital based interventions could provide needed insights into potential mechanisms and relevance for social prescribing in the absence of other data. Social-capital based interventions may share many operational similarities with social prescribing, although they are not labelled or conceptualized as such (27). For refugee populations in particular, such interventions play a crucial role in reducing isolation, fostering belonging, and enabling access to resources given the disruption to existing networks (8, 13, 17). Examining this body of literature alongside formal social prescribing research provides a necessary complementary stream of insight, helping to identify what kinds of approaches may be effective in refugee contexts.

## Objectives

This review therefore assesses evidence resulting from defined social prescribing interventions (RQ1), as well as that resulting from social-capital based interventions which are operationalized in ways comparable to social prescribing (RQ2):

RQ1: What approaches to social prescribing focused on refugee populations work, for whom, and in what circumstances?

RQ2: What additional insights can social capital-based interventions provide about which approaches with refugee populations may work, for whom, and in what circumstances?

Both questions explore the same fundamental inquiry, but draw on distinct yet complementary evidence bases. RQ1 is the primary research question; RQ2 provides contextual and inferential support where formal social prescribing evidence is absent or limited. This hierarchy reflects the anticipated scarcity of RQ1-eligible evidence rather than a reduced importance of the underlying enquiry. Where RQ2 evidence is integrated, findings should be understood as theoretically-grounded inferences about what may work in social prescribing contexts, informed by interventions sharing comparable operational logic, rather than direct evidence.

Given the anticipated scarcity of formal social prescribing evidence, this dual approach enables us to generate transferable insights by examining how similar operational approaches function across contexts. In this context, transferability refers to the applicability of mechanistic insights across intervention types that share operational features. That is, transferable insights about how and why an intervention catalyzes outcomes in one context can inform understanding of comparable approaches elsewhere, consistent with realist principles of context-sensitive knowledge transfer. We use the term “approaches” to refer to the overall intervention strategies or methods employed, which characterize through two analytical steps, defining intervention families and initial programme theories. We first identify intervention families that share operational modes to characterize distinct operational approaches observed across both evidence sources, then develop initial programme theories (explanatory statements of how mechanisms work) to explore how and why these approaches produce outcomes in specific contexts. This analytical structure allows us to synthesize evidence from both RQs to generate insights for social prescribing implementation with refugee populations. Consistent with realist methodology, answering these questions includes identifying where evidence is weak or absent, thereby establishing research priorities where current knowledge cannot adequately inform practice or policy decisions.

## Methods

### Review design

We conducted a rapid realist review, using established RAMESES publication standards for realist syntheses (31), complemented by guidance on rapid realist design (32), and more general rapid review methodology (33). The rapid realist review approach was selected because it supports theory-driven analysis of complex interventions, enabling us to ask not only whether interventions “work,” but for whom, in what contexts, and why. A rapid design was adopted to enhance timeliness, policy relevance, and feasibility, while maintaining transparency about adaptations made.

Full methodological detail, including justification of design choices and procedural specifications, is provided in the published protocol. The protocol for this review is under review and provides further methodological detail (34). Accordingly, we here summarize only the key methods used.

### Eligibility criteria

We defined inclusion and exclusion criteria using a PCC framework (participants, concept, context), which are summarized in Table 1.

**Table 1:** Inclusion and exclusion criteria.

Framework and study type	Inclusion criteria	Exclusion criteria
Participants	Refugee populations, asylum seekers, forcibly displaced persons (any age, gender, stage of settlement)	Studies where <50% of participants are refugees Studies focusing only on return/re-acculturation in country of origin Service providers without direct refugee participation Internally displaced persons were excluded from the final review as this may constitute a different experience
Concept	Social prescribing interventions: referral from health services to activities Social-capital based interventions operationalized in comparable ways (e.g., peer support, supported signposting)	Interventions confined to closed communities without intervention components Interventions are not operationalized in comparably Studies lacking sufficient methodological detail to understand mechanisms Specific exclusion cases: CBPAR/PAR focused interventions to be excluded where the only focus on the development of an intervention and not intervention delivery Digital interventions or self-help with no social component Residential interventions Studies focusing on refugee persons as mentors/befrienders, without focus on mentees/befriendees
Context	Any country context Formal search: Published between 2014–2024 Publications in English	Non-English publications
Study type	Quantitative, qualitative, or mixed-methods empirical studies Relevant grey literature (project reports, evaluations)	Purely theoretical/conceptual papers Background articles without active intervention elements

Eligible participants were refugees, asylum seekers, and forcibly displaced persons of any age, gender, or settlement stage; internally displaced persons were planned to be considered separately.

The central concept of interest was formal social prescribing interventions, as well as social-capital-based interventions operationalized in comparable ways (e.g., peer support, signposting, or community-based group activities).

In keeping with a realist approach, any study design (quantitative, qualitative, or mixed methods) and relevant grey literature were eligible, provided they contained sufficient methodological detail to enable realist synthesis. Restrictions were made that publications must be available in English and that a time filter (2014–2024) was set for the formal search, based on scoping work that suggested interventions before this period were more rarely found in the literature and contextually significantly different. No time restriction was placed on citation chasing from the texts included in full text screening, which means several relevant studies were identified from the 2004–2014 time period and included in the screening.

Specific exclusions are detailed in Table 1 [e.g., purely theoretical papers, digital only interventions without a social component, and studies where less than half of participants were refugees, for further detail see protocol (34)]. These exclusions were agreed to ensure that only empirically evaluable interventions with conceptual relevance to social prescribing were included.

### Search strategy

**Figure 1:**
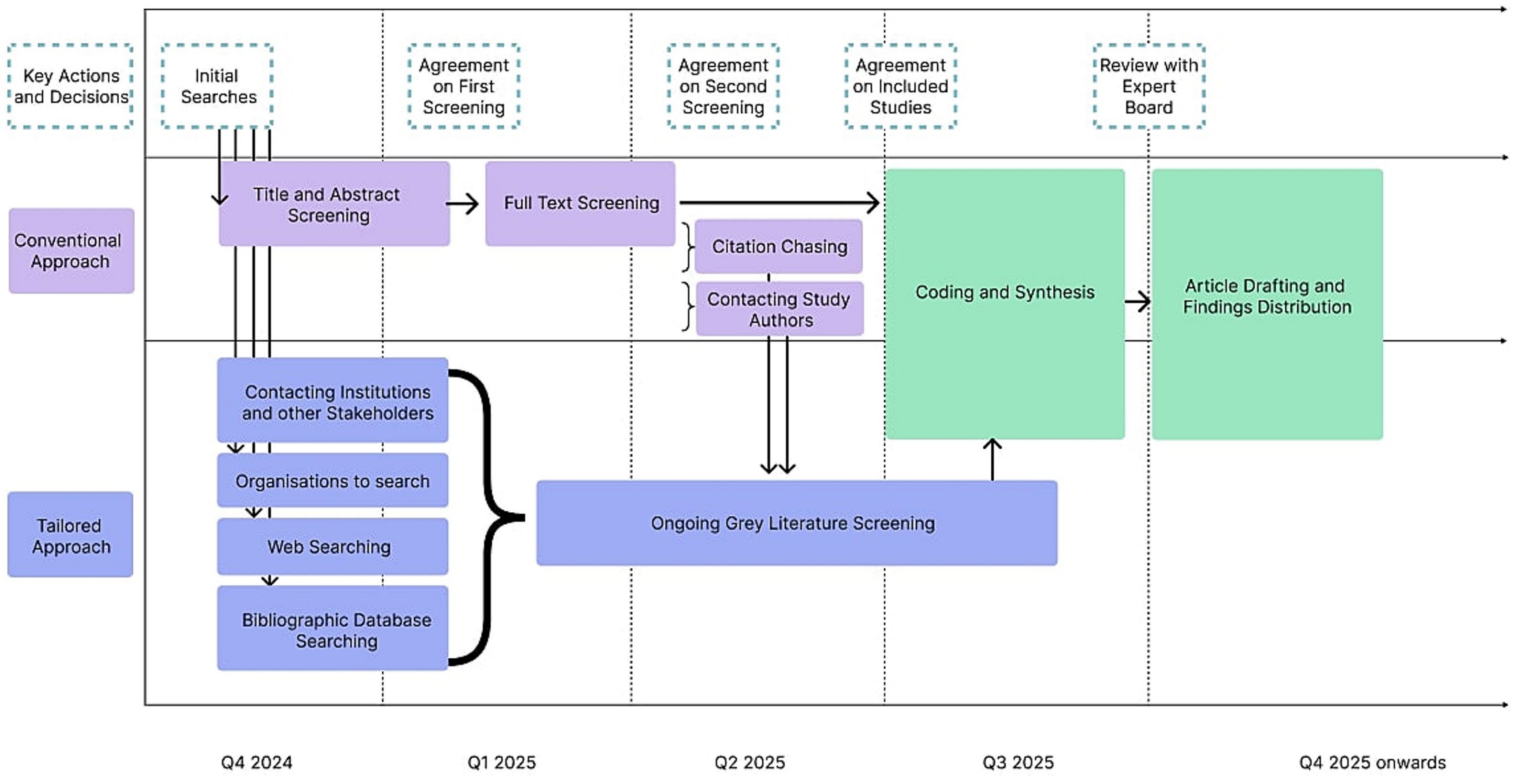
Literature strategy.

Two complementary search strategies were employed: a conventional database search and a tailored supplementary approach (see Figure 1).

The conventional search collected evidence from six major bibliographic databases (PubMed, Web of Science, Embase, CINAHL, SCOPUS, PsycInfo). Searches were conducted in October 2024 and limited to 2014–2024. Full search strings are provided in Supplementary Data Sheet 1.

As with reviews on similar topics, the tailored strategy sought to capture non-indexed material, including relevant grey literature. We searched institutional websites, contacted organisations and charities, posted requests to research networks and centres, and evaluated results from Google, Google Scholar, and an additional database, SOLO. Citation chasing was performed for all included articles at full text screening, including 56 relevant reviews, to identify additional studies. We also set up Google Alerts for ongoing updates. Stopping rules were applied when further searching no longer yielded conceptually new material (e.g., after scanning the first 500 hits of Google results).

### Study selection

All identified records were imported into Rayyan for screening, after initial hand cleaning where relevant (e.g., Google searches). A calibration exercise was first undertaken on 15% of titles and abstracts, screened in duplicate by two reviewers, to ensure consistent application of inclusion and exclusion criteria; full calibration procedures are described in the protocol (34). After calibration, the remaining records were screened by the lead investigator, with uncertain cases once more flagged for discussion with both the second and third reviewers.

**Figure 2:**
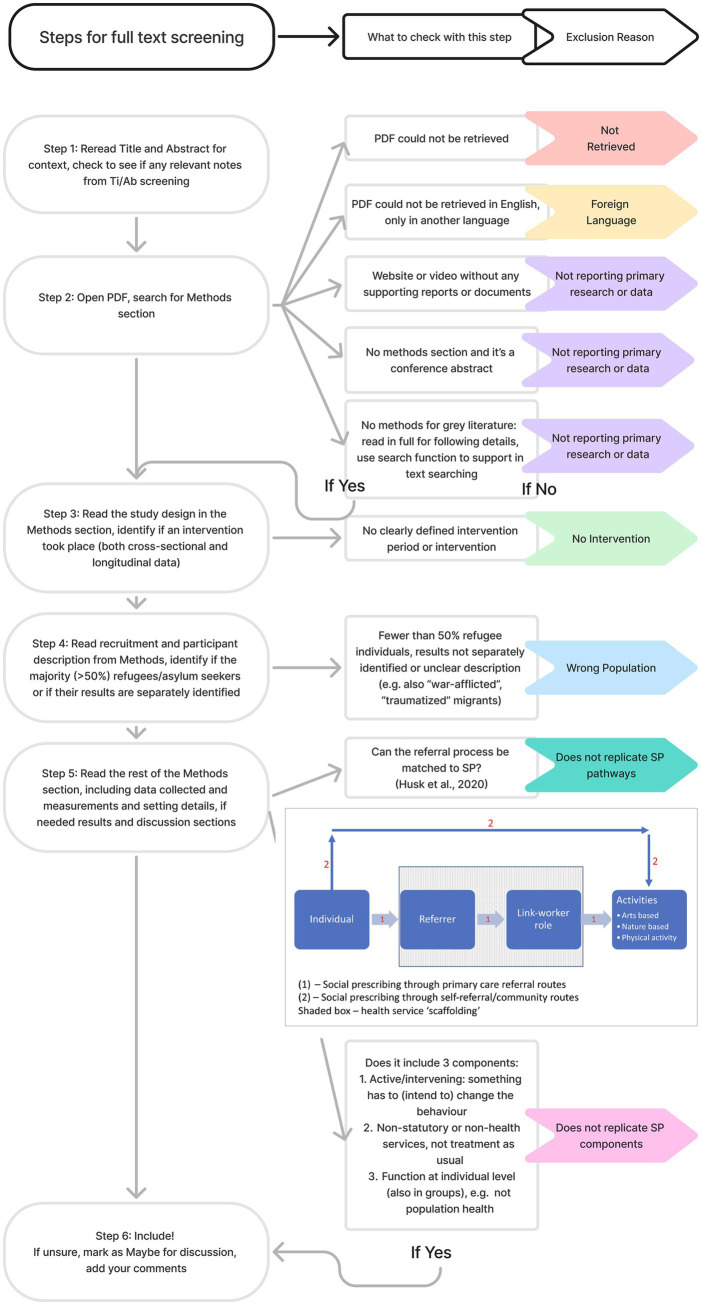
Screening tree.

Full texts were then assessed against inclusion criteria by two reviewers working independently, with additional support provided for applying criteria in uncertain cases and discrepancies resolved by consensus with a third reviewer (see Figure 2). Screening decisions were documented, and the overall selection process documented using a PRISMA 2020 flow diagram.

### Appraisal of relevance, richness, and rigour

Following completion of the screening process in Rayyan, all included studies after the full text screening were exported in Excel form. These were first appraised for relevance, richness, and rigour before the final included study set was decided, following practice guidance in how this can be assessed (23, 35, 36). Full appraisal criteria and the hybrid appraisal approach used to accommodate qualitative and quantitative evidence are described in the protocol (34).

For the purpose of this review, the key decision rule for inclusion was that studies assessed as having high or medium relevance were prioritized for final inclusion. These were defined, respectively, as directly addressing social prescribing or comparable social-capital interventions with clear mechanism descriptions, or addressing related interventions with transferable mechanistic insights. Studies assessed as having low relevance, defined as having tangential focus or limited transferability to social prescribing contexts, were excluded at this stage. Richness and rigour assessments then informed the weight given to each study in synthesis, rather than functioning as binary inclusion thresholds. Assessments were conducted by one reviewer and verified by a second reviewer. Discrepancies were resolved through discussion and a third reviewer consulted where needed.

### Data extraction and management

The included records were first organized and grouped into relevant study clusters (i.e., studies describing the same intervention or project were grouped together). A further hand search took place to assess whether any other supporting documentation existed to complete the data picture for each study cluster (e.g., linked protocols, separately published results etc.) and where identified, these were then grouped together.

A structured extraction template was then used to record study characteristics, context, mechanisms, outcomes, and relevance to the review questions from all study clusters, with codes also identified from results from a series of expert interviews (37). This was completed by the lead reviewer and checked by the second reviewer.

### Data synthesis

Synthesis followed realist logic principles, operationalized through five iterative steps (38):

1. Juxtaposition of sources: examining how different studies contributed complementary insights.
2. Reconciliation of findings: exploring where results diverged and identifying contextual explanations.
3. Adjudication: giving greater weight to studies with greater relevance or rigour.
4. Consolidation: combining multiple sources to reinforce emerging mechanisms.
5. Situating: considering transferability across contexts and populations.

Following realist synthesis methodology (31, 38), we conducted our analysis in two stages. We first mapped interventions into “families” i.e., groupings of studies that shared operational modes (such as peer facilitator models, comprehensive barrier-reduction models) to identify patterns in how interventions were structured and delivered. Second, we developed initial programme theories, which are explanatory statements standard in realist evaluation (31, 38) that articulate how specific mechanisms are activated in particular contexts to produce outcomes, structured in CMO configurations (context-mechanisms-outcome). In order to maintain clarity, we express our CMO configurations in the form of If-Then statement, which we feel allows us to articulate meaning in ways that make more sense [e.g., if (context), then (mechanism), leading to (outcome)] (18, 36). We adopted an abductive approach: initial programme theories were developed deductively from existing literature and the intervention family patterns identified, then inductively refined as evidence from included studies was synthesized and tested against extracted data. Final programme theory wording was finalized through discussion with the expert advisory board (34).

### Stakeholder engagement

Stakeholder input was sought from seven experts, principally through online meetings at two key stages to ensure relevance and policy applicability, in line with Touzel et al. (34). An external advisory board, composed of academics, practitioners, and community representatives, contributed to question formulation, priority-setting in the extraction, sense-checking of findings, and discussion of initial programme theories (see Acknowledgements).

We note here that the composition of the advisory board, who primarily come from minority-world, high-income country contexts, and the positionality of the review team as academic researchers without direct lived experience of forced displacement may have shaped the framing of programme theories and the prioritization of certain mechanisms over others. These are acknowledged as limitations of the current evidence landscape rather than as correctable methodological flaws, but are relevant to the cautious interpretation of findings, particularly considering majority-world and intersectional population contexts.

### Rapid review process

Several methodological adaptations were made to align with rapid review and rapid realist review principles (32, 33). These are summarized in Supplementary Data Sheet 2 for full transparency about methodological choices made and their trade-offs.

## Results

### Overview of search results and study selection

**Figure 3:**
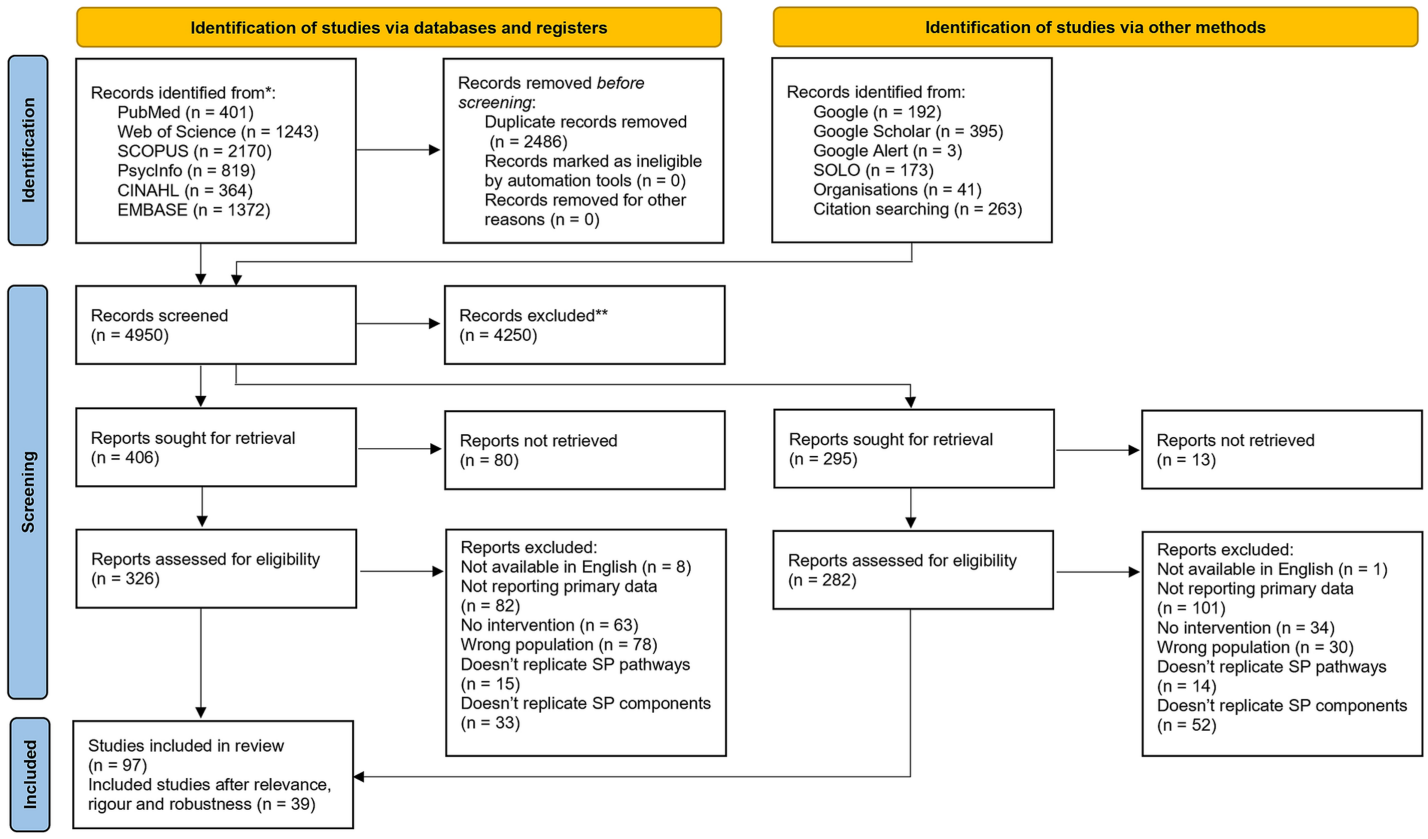
PRISMA flowchart.

A full list of records at each screening stage, Supplementary Table 1, a visual summary guide for screening decisions, Figure 2, and the PRISMA flowchart detailing the screening flow of records, Figure 3, are provided. Our search strategy yielded a total of 7,436 records, 6,369 from the conventional approach (databases searched) and 1,067 from the tailored approach (grey and informal literature searches). 2,486 records were excluded as duplicates with 4,950 records undergoing title and abstract screening. A calibration exercise was conducted on 15% of all records, which were screened by two reviewers and discrepancies discussed as a group with a third reviewer. The remaining 85% were screened by one reviewer, with 701 records progressing to full text screening. Full texts were screened by two reviewers independently with blinding, with final discrepancies discussed and resolved in agreement with the same third reviewer as for the title-abstract screening. After excluding records that could not be retrieved, 608 records were screened as full texts with 97 proceeding for inclusion. These 97 records were then assessed for relevance, richness, and rigour using a bespoke appraisal criterium based on previous review approaches and supported by CASP, NIH and MMAT assessment tools (23, 35, 36). Records that were found to have low relevance were not prioritized for further consideration, with all others considered on a one-to-one basis considering both conceptual richness and study rigour through exchange between two reviewers. Following this step, 39 studies were included in the final synthesis, organized into 34 study clusters where multiple studies related to the same intervention or programme were grouped together.

### Characteristics of included studies

Table 2 provides an overview of the included studies, detailing study cluster, constituent design, intervention or programme focus, geographical and setting contexts, populations involved, study designs used, outcomes, and summary findings for relevance, richness, and rigour appraisals. Full extraction details are available in Supplementary Table 2. Data was extracted by one reviewer and cross-checked by the reviewer who had also conducted full-text screenings.

**Table 2:** Included studies (extraction details are available in Supplementary Table 2).

Associated references for study clusters (SC): SC1 (51, 52); SC2 (39); SC3 (59, 60); SC4 (64); SC5 (44); SC6 (78); SC7 (79); SC8 (40); SC9 (61); SC10 (53, 54); SC11 (62); SC12 (55); SC13 (80); SC14 (65); SC15 (45); SC16 (73); SC17 (43); SC18 (56, 57); SC19 (81); SC20 (41); SC21 (63); SC22 (72); SC23 (66); SC24 (71); SC25 (67, 68); SC26 (46); SC27 (58); SC28 (69); SC29 (47); SC30 (42); SC31 (70); SC32 (48); SC33 (49); SC34 (50).

The included evidence base reflected substantial heterogeneity across multiple dimensions, as anticipated given the exploratory nature of this review and the guiding research questions. A summary of study heterogeneity across key dimensions is provided in Box 1. Only 6 study clusters described pathways and mechanisms which aligned closely with social prescribing (and only 1 study self-identified as a social prescribing intervention). The majority of included evidence was identified by the conventional database searches, as opposed to only 3 studies that were identified using the tailored approach. This is relevant to final findings, as 2 of those 3 studies aligned to social prescribing and it is possible that yet further valuable evidence may be available outside peer-reviewed literature contexts (particularly in the form of service evaluations and reports). In summary, the majority of included studies represent peer-reviewed publications which respond to RQ2. Our review results should therefore be understood as inferred evidence predominantly from social-capital based interventions applied to social prescribing contexts.

BOX 1 **Summary of heterogeneity across included studies (*n* = 39 studies; 34 study clusters).**

**Table:** 

Dimension	Summary (% of SCs)
Publication dates	2005–2024; 69% from 2019–2024
Geography	USA (38%), Europe (32%), Australia (12%), Canada (9%), with limited evidence from majority world settings (e.g., Lebanon and South Africa, 9% total)
Programme settings	Community centres (53%), municipal or school centres (24%), home-based (12%), health clinics (6%), online (3%) or sports centres (3%)
Legal status reporting	Unreported in 62% of SCs; inconsistently defined where reported, e.g., participants described as “recently resettled,” “newly arrived,” without consistently specifying time since arrival, legal determination status, or settlement stage
Age groups	Full life course representation, including youth or children (12%), adults (70%), and intergenerational families (18%)
Ethnic/national focus	Multiple studies used only generic terms like “adult refugees” without further specification or with mixed ethnic groups; where specified, ethnic groups included Syrian (12%), Bhutanese (12%), Somali/Somali Bantu (9%), Sudanese (9%) participants
Specified subgroups	Specific populations were addressed through gender segregated (35%) or ethnic groups (41%), with further specified subgroups including torture or war survivors (18%), refugee mothers (12%), and unaccompanied refugee youth (6%)
Intervention duration	Spanned 2 weeks to 28 months; 38% fell within a 6–12-week range
Leadership models	Emphasized peer and community leadership including through peer/community-led models (35%), community-led staff models (29%), and co-design approaches (47%)
Study design	Predominantly qualitative (65%) and mixed-methods (35%); limited quantitative evidence; pre-dominantly cross-sectional data collection

Delivery formats ranged from structured, manualized protocols (particularly in trauma-focused psychosocial interventions) to flexible, participant-responsive approaches in community-based programmes. The theoretical underpinning of programmes was diverse, spanning trauma-informed care models [e.g., SC2 (39)], creative therapies [e.g., SC8 (40)], livelihood development and employability approaches [e.g., SC20 (41)], befriending services [e.g., SC30 (42)], and community hosting [e.g., SC17 (43)], reflecting the multidimensional nature of person-centred refugee health and wellbeing needs.

This methodological distribution reflects the exploratory nature of the field and the nature of refugee-focused community interventions, where co-production and participant voice are often central to intervention design and where understanding process and experience is prioritized alongside outcomes. These variations are therefore interpreted as reflecting the genuine diversity in how refugee-focused community support is conceptualized and delivered across different contexts and were treated analytically as meaningful contextual variation.

However, this methodological profile has direct consequences for realist inference and policy translation. The predominance of qualitative and cross-sectional designs combined with inconsistent reporting and mixed population characteristics limits generalisability, representativity, and causal interpretations. CMO configurations derived from this evidence primarily describe plausible explanatory pathways rather than confirmed causal relationships. Furthermore, their translation into policy recommendations requires acknowledgement that implementation contexts (particularly in under-represented majority world or population group settings or with alternative methodologies) may activate different mechanisms or surface additional barriers not identified in the current evidence.

### Analysis approach for developing intervention families

**Figure 4:**
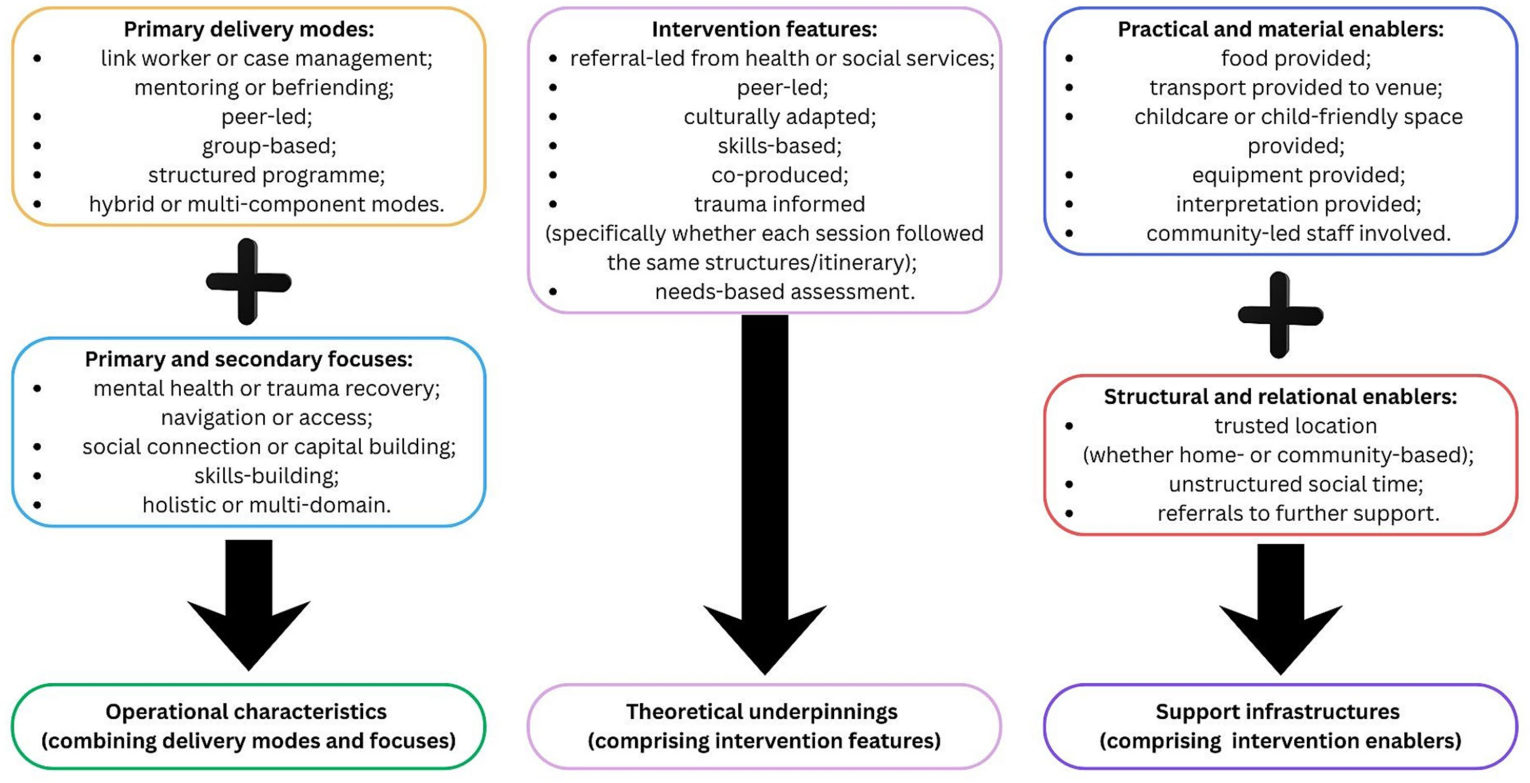
Characteristics mapping.

Following initial data extraction and consistent with realist methodology, we mapped included studies according to their operational characteristics, theoretical underpinnings, and support infrastructures (see Figure 4). In order to begin analysis, all interventions were mapped for their primary delivery modes, primary and secondary focuses, features, and enablers. Primary delivery modes were allocated based on delivery and intervention focuses, finally comprising: link worker or case management; mentoring or befriending; peer-led; group-based; structured programme; or hybrid or multi-component modes. Focuses were grouped into: mental health or trauma recovery; navigation or access; social connection or capital building; skills-building; and holistic or multi-domain. The majority of study clusters, e.g., the groupings of studies focused on the same intervention, had multiple focuses (*n* = 28; 82%). Based on extraction material and previous exchange with expert advisory board members, intervention features and enablers (both practical or material supports and structural or relational supports) were assessed. Features that were assessed across all study clusters included whether the intervention was: referral-led from health or social services; peer-led; culturally adapted; skills-based; co-produced; trauma informed (specifically whether each session followed the same structures/itinerary); and needs-based assessment. Practical and material enablers included: food; transport; childcare; equipment; interpretation; and community-led staff. Structural or relational enablers included: trusted location (whether home- or community-based); unstructured social time; and referrals to further support. Further detail and summary tables can be found in Supplementary Table 3, including interactive summaries within the data consolidation sheet.

**Figure 5:**
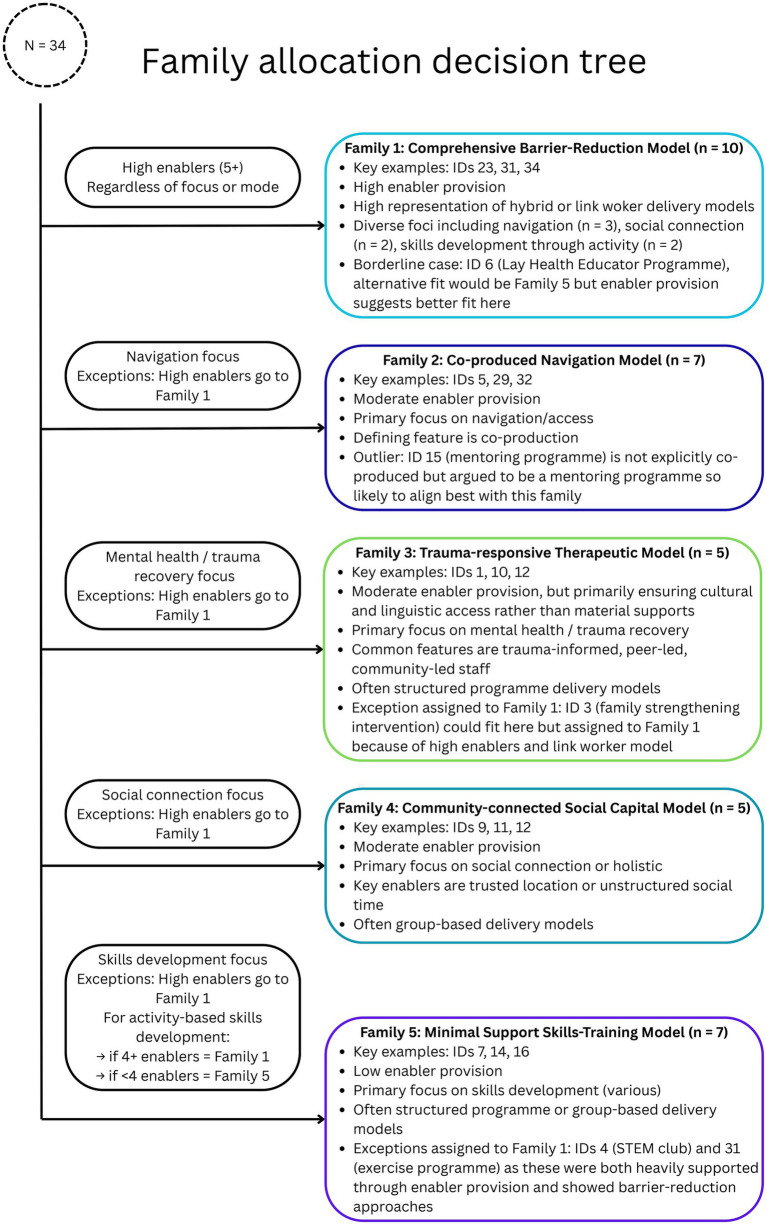
Family allocation decision tree.

The intervention family mapping process, guided by the decision tree presented in Figure 5, enabled us to identify distinct groupings of interventions with distinct operational approaches, e.g., that function comparably despite potentially different labels or local adaptations. This family-based approach reflects the rapid realist review emphasis on identifying intervention-outcome patterns within specific parameters rather than developing fully transferable theories across all contexts.

**Figure 6:**
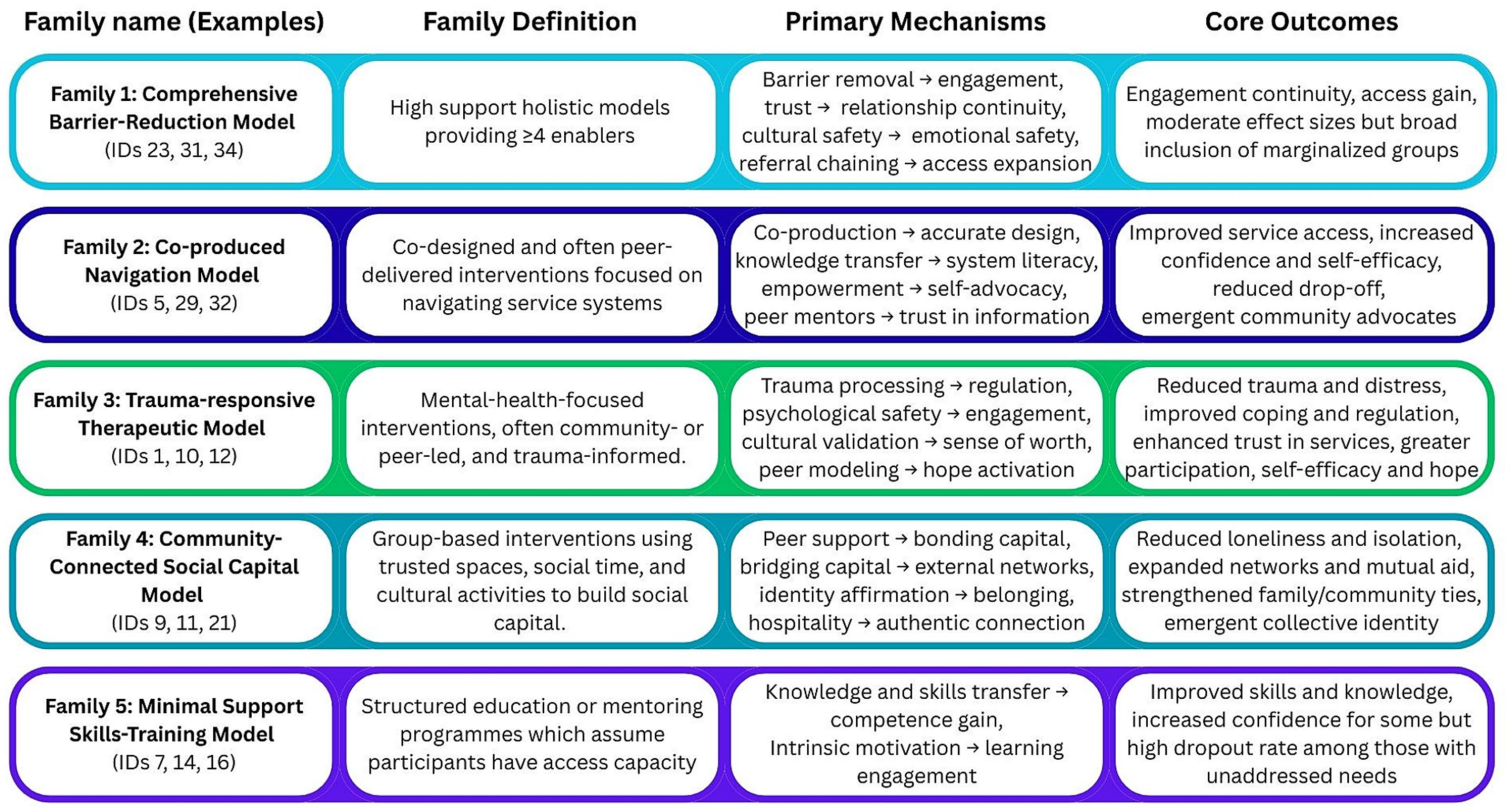
Families overview.

We identified five intervention families representing distinct operational approaches during this analytical process, as illustrated in Figure 6. These families included: (1) comprehensive barrier-reduction model; (2) co-produced navigation model; (3) trauma-responsive therapeutic model; (4) community-connected social capital model; (5) minimal support skills-training model. Family membership was determined primarily by operational mechanisms rather than intervention labels, acknowledging that similar approaches may be conceptualized differently across contexts or that programmes labelled identically may operate through different mechanisms (e.g., study 33 and 34 comparison).

This family-based organization served as the foundation for subsequent development of initial programme theories, enabling us to identify patterns in how similar intervention approaches activated mechanisms within particular contexts to produce outcomes. The intervention families are explicitly not mutually exclusive—within the identified studies, some interventions incorporated elements of multiple families and it was clear from discussion with the expert advisory board that in reality many community organisations may integrate multiple models in their service delivery.

### Synthesis approach for initial programme theory development

Following the organizational mapping of studies above, our synthesis proceeded through iterative stages of juxtaposing, reconciling, adjudicating, consolidating, and situating evidence to construct initial programme theories (explanatory CMO statements as described in Methods). The 23 initial programme theories spanned cross-cutting statements, family-specific statements, context statements, and additional statements (particularly focusing on elements that could provide key insights for social prescribing, e.g., referral pathways into interventions/programmes). As evidence from the included studies was synthesized, these theories were inductively refined and tested against the extracted data (see Supplementary Tables 3, 4). Where concepts such as safety and co-production recur across multiple programme theories and intervention families, this is analytically intentional. Consistent wording across contexts reflects that the same underlying mechanism has been identified operating across distinct contexts, which is itself a cross-cutting finding. These 23 statements were appraised for relevance to RQ2, where evidence would provide meaningful insights into mechanisms potentially relevant to social prescribing delivery with refugee populations.

Each programme theory was assessed for evidence strength based on two key dimensions. First, pattern evidence evaluated the consistency and breadth of patterns observed across studies. Strong pattern evidence indicates clear consistent patterns across multiple study clusters, moderate indicates observable patterns with some variation, weak indicates minimal or inconsistent patterns. Second, mechanism evidence evaluated the clarity and empirical support for the underlying mechanisms. Strong mechanism evidence indicates well-described, empirically demonstrated mechanisms, moderate indicates mechanisms that are inferred from data but not directly tested, weak indicates primarily theoretical or logical inferences without empirical corroboration. Overall evidence strength combines these dimensions: strong theories show clear patterns across multiple studies with well-described mechanisms; moderate theories show observable patterns with limited mechanism evidence; weak theories show minimal evidence, are primarily theoretical, and represent research needs requiring validation. Complete evidence mapping is provided in Supplementary Data Sheet 3.

A final shortlist of 15 statements were taken into discussion with our expert advisory board (see Supplementary Data Sheet 4). Where evidence was limited, theories were flagged as identified research needs for validation with the expert board. Conversations with the expert board therefore focused on iteratively improving the wording of the final insight statements, sense-checking these statements matched experience or expectations from experts, and validating perceived priority for identified research needs.

**Figure 7:**
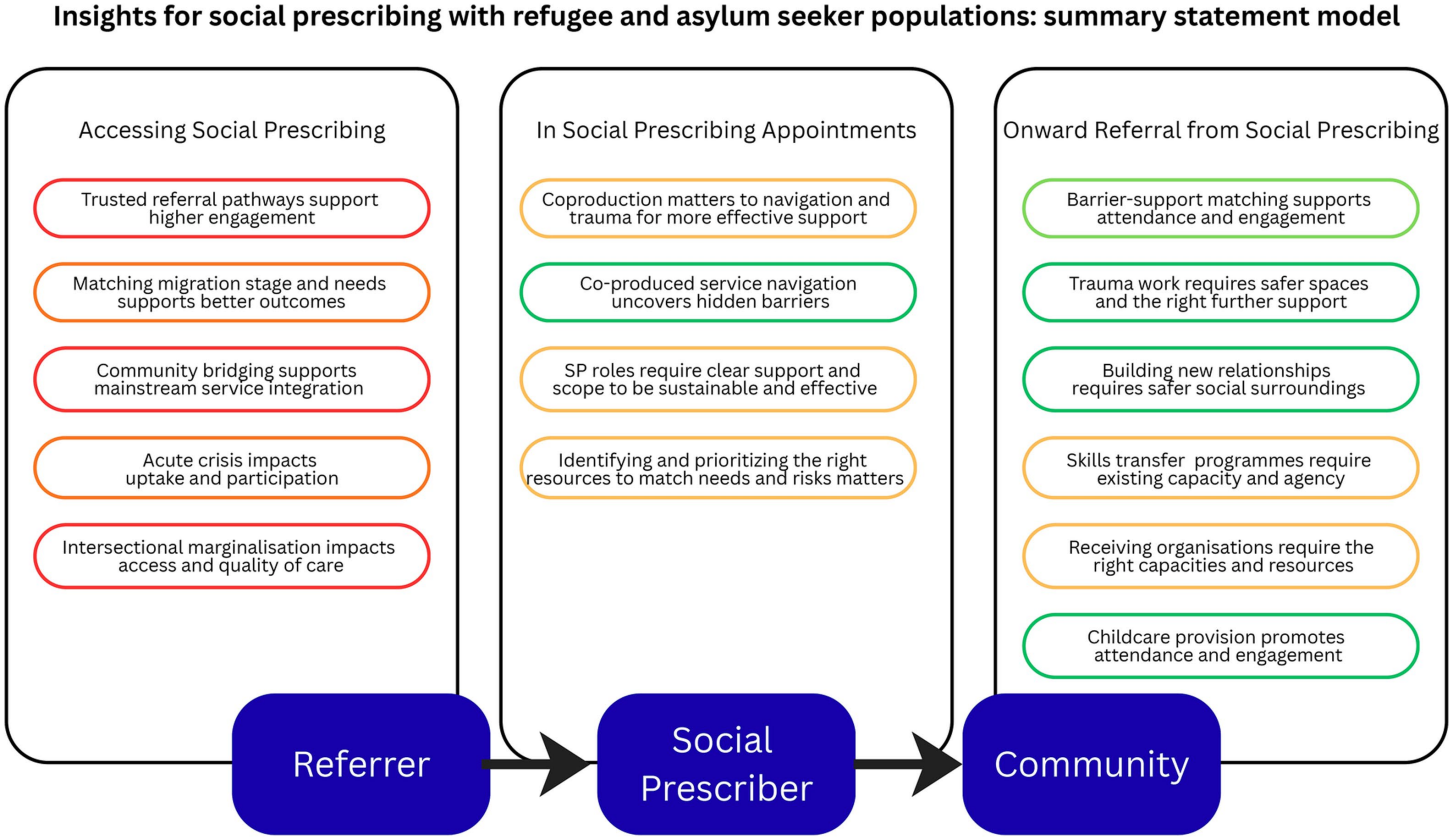
Summary statement model.

Through this analytical process, 15 initial programme theories were generated and agreed with the expert advisory board. These are organized around key dimensions of social prescribing delivery: referral and routes into social prescribing is grouped into “accessing social prescribing”; supporting initial uptake, engagement, and meeting specific population needs is grouped into “in social prescribing appointments”; and supporting onward referral and participation grouped into “onward referral from social prescribing.” These programme theories, presented in Table 3, represent the synthesized understanding of insights into what approaches may work, for whom, and under what circumstances, drawn primarily from social-capital based interventions operationalized in comparable ways to social prescribing. Throughout the theory narratives below, mechanisms are described as “inferred” or “posited” to denote theory-informed extrapolations from social-capital intervention evidence to social prescribing contexts (also given the scarcity of formal social prescribing evidence). Each programme theory is grounded in evidence from multiple sources across the intervention families, with the strength and consistency of supporting evidence detailed in Supplementary Data Sheet 3. For ease-of-reading, programme theories are here formulated positively: they indicate under what circumstances social prescribing may work (rather than may not work); SP refers in all cases to social prescribing, SPs refers in all cases to social prescribers (which can be substituted for preferred role name, e.g., link workers). These are visually summarized by title in Figure 7.

**Table 3:** Summary of programme theories.

Phase	Title	Programme theory	Evidence
Accessing social prescribing	Trusted referral pathways support higher engagement	*IF* refugees and asylum seekers are referred from trusted persons or organisations to SP or from SP to community organisations through supported referrals with appropriate accompaniment for that individual *THEN* that recommendation helps build trust *RESULTING IN* higher initial engagement than with unsupported referrals	Very weak—identified research priority
	Matching migration stage and needs supports better outcomes	*IF* refugees and asylum seekers are referred to SP or from SP to community organisations based on their needs and stage of migration (e.g., newly arrived, socially/emotionally stabilized, settled) *THEN* the referral aligns with the right timing to prompt engagement *RESULTING IN* better outcomes than mismatched timing (e.g., “not the right time”)	Weak—identified research priority
	Community bridging supports mainstream service integration	*IF* refugee-serving community organisations act as empowering bridges for refugees and asylum seekers to access SP or mainstream health services *THEN* they benefit from this facilitation that supports their access *RESULTING IN* greater long-term service integration and engagement	Very weak—identified research priority
	Acute crisis impacts uptake and participation	*IF* refugees and asylum seekers are in acute crisis (e.g., homelessness, extreme poverty, imminent deportation or removal, change in status, acute mental health crisis) *THEN* even well-designed non-crisis services or community organisations may not be appropriate for them *RESULTING IN* likely risk of non-participation or non-attendance	Weak—identified research priority
	Intersectional marginalization impacts access and quality of care	*IF* GP services, social prescribing services, and community services are ready for and responsive to the specific needs of multiply-marginalized refugees and asylum seekers *THEN* these individuals are more likely to access and benefit *RESULTING IN* greater equity of access and quality of care	Very weak—identified research priority
In social prescribing appointments	Coproduction matters to service navigation and trauma disclosure for more effective support	*IF* SP appointments involve navigation of services and/or trauma disclosure with refugee and asylum seekers *THEN* co-production of a wellbeing plan and “peer” connection help recognize refugee lived expertise, bridge knowledge gaps, and individuals feeling “seen” and “heard” *RESULTING IN* more effective support, meaning that SPs’ empathy, refugee-specific insight and potentially their shared personal characteristics matter to outcomes	Moderate
	Co-produced service navigation uncovers hidden barriers	*IF* SP appointments focusing on navigation of services with refugees and asylum seekers are co-produced in a way where SPs actively create space for their input *THEN* they can share their culturally specific needs and suggest strategies that genuinely fit their context *RESULTING IN* greater chance of navigating barriers that SPs did not know about	Strong
	SP roles require clear support and scope to be sustainable and effective	*IF* SPs receive training, supervision, and compensation, with clearly defined roles (e.g., addressing navigation of services, trauma disclosure, health literacy) *THEN* they can draw on their skills and experience while reducing risk of burnout and role confusion, *RESULTING IN* more sustainable effective roles in SP	Moderate
	Identifying and prioritizing the right resources to match needs and risks matters	*IF* SPs can identify and prioritize the right resources for the needs and risks in the appointment (e.g., service knowledge for navigation of services or relational connection for trauma disclosure) *THEN* relevant further supports need to be in place (e.g., interpretation, referrals onwards) *RESULTING IN* positive outcomes	Moderate
Onward referral from social prescribing	Barrier-support matching supports attendance and engagement	*IF* SPs are supporting refugees and asylum seekers experiencing multiple barriers who would like to be referred onwards to community organisations *THEN* referrals can be made to community organisations with the capacity to meet their needs through addressing specific barriers (e.g., interpretation, childcare, trusted location, transport) *RESULTING IN* them being more likely to attend and engage	Moderate—strong
	Trauma work requires safer spaces and the right further support	*IF* SPs can connect refugees and asylum seekers with community organisations that use trauma-informed approaches, peer leadership, and are culturally appropriate *THEN* these can create safer spaces where participants feel heard and validated with the right further support (e.g., childcare, interpretation, trusted location, community-led staff) *RESULTING IN* the potential to support in trauma processing and recovery	Strong
	Building new relationships requires safer social surroundings	*IF* SPs can connect refugees and asylum seekers to community organisations that provide trusted locations and social exchange where they feel safe *THEN* the sense of physical and psychological safety in these social surroundings makes new connections possible *RESULTING IN* increased openness, creating the potential for forming positive new relationships	Strong
	Skills transfer programmes require existing capacity and agency	*IF* SPs can connect settled refugees and asylum seekers who have existing agency and capacity with training programmes which include minimal further support or accompaniment *THEN* these individuals can benefit through learning or improving skills *RESULTING IN* scalable and cost-effective skills transfer programmes	Moderate
	Receiving organisations require the right capacities and resources	*IF* community organisations receiving social prescribing referrals have the capacities and resources they require (e.g., funding/logistics; co-production infrastructure; trauma-informed culture; trusted spaces) *THEN* they can deliver activities or services as intended *RESULTING IN* achieving outcomes	Moderate
	Childcare provision promotes attendance and engagement	*IF* SPs engage with women with young children *THEN* connecting them with community organisations that provide childcare or spaces where children are welcome removes caregiving barriers *RESULTING IN* greater likelihood of mothers’ attendance and engagement	Strong

### Initial programme theories with the strongest evidence

Four initial programme theories demonstrated strong evidence (clear evidence across multiple studies with well-described mechanisms) and one programme theory demonstrated moderate—strong evidence across multiple study clusters. These warrant further elaboration for their insights and general implications for social prescribing implementation with refugee populations. Narrative depth varies across theories in proportion to the volume and richness of supporting evidence; theories drawing on larger study cluster sets or with richer mechanistic detail are discussed more extensively, reflecting the evidence base.

#### Co-produced service navigation uncovers hidden barriers

##### Evidence strength: strong|evidence: intervention family: 2 (co-produced navigation)|social prescribing phase: appointments

This initial programme theory is primarily described in seven navigation-focused interventions within family 2 (SCs: 2, 5, 15, 26, 29, 32, 33) (39, 44–49), with 86% stating explicit co-production. The one exception, SC15 (45), represents a mentoring initiative which arguably may have involved co-production. The theory is also supported by evidence from SC34 (50), also primarily focusing on navigation and access with explicit co-production, which belongs to family 1 but is an extension of the programme format first trialled in SC33 (49).

The theory posits that when social prescribing appointments focusing on service navigation are coproduced with refugees and asylum seekers, with space being actively made for their input, social prescribers can identify individual-specific barriers that would otherwise remain hidden. It may be particularly relevant during early resettlement phases but also later as a recurring need, whenever individuals lack familiarity with the relevant host country health, education, and social service systems. It may have less relevance for settled refugees who possess existing system knowledge, capacity, and agency. The CMO configuration is inferred to operate as follows: In contexts where refugees and asylum seekers require support with navigating complex service systems, co-production activates cultural brokerage mechanisms. These mechanisms enable bidirectional knowledge exchange where refugees’ expertise reveals system barriers unknown or invisible to professionals, while simultaneously building individual refugees’ navigation capacities. The resulting outcomes include more effective barrier identification, developing appropriate strategies in response, enhanced service access, and improved navigation success compared to solely professional-led navigation alone without coproduction.

Evidence from the synthesis demonstrates consistent patterns, with the presence of bilingual and bicultural workers emerging as an important contextual contingency for the theory’s application. SC5 (44), a community liaison workers study, exemplifies this mechanism, particularly for the bidirectional benefits of knowledge exchange with community members being empowered to access services and service providers responded with improved cultural understanding in delivery. In contrast, SC15 (45) which describes a mentoring initiative which paired asylum seekers with mentors from the host community but without explicitly referencing that this was co-produced, also describes similar patterns. Mentees reported greater resources to access services while mentors reported personal enrichment through greater solidarity, valuing insights into mentees’ circumstances, and using their increased knowledge to address attitudes towards refugees in their wider social circles, clearly highlighting the value of bidirectional exchange.

#### Trauma work requires safer spaces and the right further support

##### Evidence strength: strong|evidence: primarily intervention family 3 (trauma-responsive therapeutic model)|social prescribing phase: appointments

Five mental health and trauma-focused interventions from family 3 (SCs 1, 10, 12, 18, 27) (51–58) provide strong evidence for this programme theory. A further comparable intervention with a mental health focus, SC3 (59, 60) from family 1, also shared several of the key features (peer-led, culturally adapted, with community-led staff). Within the family 3 evidence, 80% of intervention employed trauma-informed approaches (specifically using the same structure for sessions delivery to establish expectations and create sense of safety for participants). A further 60% incorporated peer leadership, 40% drew on community-led staff, and 80% used culturally adapted content in delivery. There was clear feature synergy between trauma-informed and peer-led delivery, trauma-informed and culturally adapted delivery, and peer-led and community-led staff delivery.

Here the mechanism configuration is inferred to activate through “shared witness” dynamics, where peer leaders with lived experience of forced migration establish a space where experiential knowledge can be validated. Outcomes documented across these interventions include participants reporting feeling heard and validated in their experience, increased willingness to engage in trauma processing, and measurable improvement in mental health indicators. However, there are likely important contextual factors that significantly moderate effectiveness. The interventions likely require stabilized living conditions, that participants are socially and emotionally stable before they are able to engage with this content (exception to this is seen in SC27 (58), described below). Consistent attendance is also critical to outcomes and supported trauma work, with session structure providing predictability that enhances psychological safety.

Evidence indicated that approaches to operationalizing trauma work varied while maintaining core structural elements. For instance, SC1 (51, 52), a family-based social emotional learning intervention, emphasized the importance of establishing predictability through clear session architecture, where participants acknowledged the intervention to have created a safe social bonding space during COVID-19. In comparison, SC12 (55), an after-school group programme integrating African drumming in each structured session with a peer “pyramid” mentoring structure, highlighted in learnings that evidence-based trauma interventions should be adapted to be culturally responsive and that peer mentoring created “reciprocal healing,” as graduates expressed the desire to keep supporting others. A compelling example is also given by SC27 (58), a drama therapy intervention with women in a refugee camp setting. This intervention demonstrated the feasibility of trauma-informed approaches using the same structure for sessions even within constrained environments, through cultural adaptations to therapeutic practice for traditional storytelling methods and movement exercises. Despite ongoing environmental instability in a camp setting, participants noted that they had regained self-confidence and had experienced it as empowering to share their stories with peers to reconcile with painful memories. These examples collectively suggest that the mechanism of safer social spaces operates through multiple reinforcing channels—structural predictability, experiential validation, and cultural resonance—rather than through any single intervention component.

#### Building new relationships requires safer social surroundings

##### Evidence strength: strong|evidence: intervention family 4 (community-connected social capital model) | social prescribing phase: onward referrals

Family 4 interventions (SCs 9, 11, 17, 21, 30) (42, 43, 61–63) demonstrate that social capital building requires specific environmental conditions, with 60% explicitly utilizing trusted community locations. The exceptions, SCs 17 and 21 (43, 63), were either indeterminate or mixed, e.g., SC17 describes a nationwide hosting programme with local organisations, but it was not possible to determine in all cases that these would represent culturally appropriate community spaces, whereas SC21 was delivered in community centres and churches but also schools. Therefore, in no case was the setting clinical or institutional (in the sense of university or government departments).

The programme theory posits that physical and psychological safety within social spaces creates the necessary conditions for positive relationship forming for refugees. The mechanism operates through spatial and relational safety creating an environment that supports greater openness and thereby the potential for connection. In terms of outcomes, both quantitative and qualitative evidence demonstrates measurable improvements in social connectivity. Although longitudinal impact was more rarely reported, some studies indicated ongoing improvement in relational outcomes beyond individual connections made and beyond the intervention period. This was seen in SC9 (61), a befriending and bridging initiative which demonstrated that participants had made an average of 3.6 friends with post-intervention reporting indicating that they had retained an increased number of friends. Similarly, SC11 (62) describes a mothers befriending programme where the importance of a strengths-based approach is noted: participants wanted to independently continue the group after the intervention and to maintain friendships, a huge relational step from believing themselves to have little to offer to one another and the community.

Evidence suggests that the presence of unstructured time is important, allowing relationships to develop organically rather than through programme facilitation. Two included study clusters explicitly included unstructured social time and operationalized this differently. In SC21 (63), a peer-support group project, reflections emphasized the importance of both allowing participants to organically create connections but also to provide targeted activities and topical information sessions, creating the necessary social environment for group bonding and mutual support. This community capacity-building was seen on an individual-level through peer support in shared experiences, disconfirming the belief that individuals are alone in their suffering, and in time through a parasocial ripple effect where refugees are able to spread knowledge from the programme to others who were not involved. In SC30 (42), a befriending scheme between refugees and asylum seekers and local community members, the importance of being together, reciprocity and good humour with regular and committed contact was described as crucial to bridge cultures, address stereotypes and work through misunderstandings. Here ripple effects were also noticed particularly for the befrienders being better able to challenge dominant negative discourses in personal circles. Both SCs’ focuses on capacity-building and implicit strengths-based reciprocal approaches indicate the potential individual growth through social connection improving self-worth and self-recognition as potential contributors.

#### Childcare provision promotes attendance and engagement

##### Evidence strength: strong|evidence: cross-cutting, notably family 1 (comprehensive barrier-reduction model)|social prescribing phase: onward referrals

Twelve studies providing childcare or spaces where children were welcome establish this as an important practical enabler (SCs 1, 3, 4, 8, 14, 17, 20, 23, 25, 28, 29, 31, 34) (40, 41, 43, 47, 50–52, 59, 60, 64–70). Within family 1 interventions, 80% provide childcare, demonstrating a strong pattern with engagement of multiply-marginalized refugee women and families. Although this theory receives less extended discussion than others in this section, this reflects the relative directness of its mechanism rather than any lesser importance.

This mechanism operates at both practical and psychological levels. Practically, childcare removes the immediate barrier of caregiving responsibilities while psychologically, knowing children are safe and entertained enables full caregiver participation. A key illustrative SC in this regard is SC4 (64), a STEM club for teenage refugee girls. In this example, the authors discuss the implications of refugee childhood and youth denial for teenage girls as an atmospheric wall, where girls both noted the lack of toys in their community centre and were also only able to attend if they also brought their younger siblings with them. On the psychological and developmental level, girls chose to make toys and handbags as a first activity, given that these were needed in their community. Through the process, they also established new circles of kinship, with girls taking responsibility for communal babysitting and seeking to engage their charges in making these new artefacts. This process of community-building in a space where children were welcomed and involved contributed to giving the girls new purpose and establishing rightful presence within the community centre by pushing back on assumptions of what mattered to them (e.g., school backpacks and practical clothes dominating donations).

#### Barrier-support matching supports attendance and engagement

##### Evidence strength: moderate—strong|evidence: cross-cutting|social prescribing phase: onward referrals

All 34 study clusters contribute evidence to this moderate-to-strong programme theory, with clear stratification patterns in enablers across intervention families. The theory articulates that support intensity must align with population barriers to enable participation and engagement. The evidence suggests a clear inverse relationship between population barriers and service accessibility without appropriate enabler provision (considering both practical and structural/relational supports).

Family 1 interventions demonstrate the highest enabler provision (mean 5.5, range 4–8), serving populations experiencing multiple barriers including recent arrivals, war and conflict survivors, those in poverty and or with caregiving responsibilities. In contrast, family 5 interventions provide minimal enablers (mean 2.1, range 1–3) serving more established populations with existing capacity or agency. Key comparative employability skills examples come from similar settings in Denmark, where SC8 (40) describes a community garden project for Syrian refugees (family 1, including social time, referrals onwards, food, childcare, equipment, interpretation) and SC24 (71) a skills-building programme based in an eco-village around gardening activities (family 5, including food and equipment).

The differential outcomes between these interventions illustrate how critical barrier-support matching may be in practice. In the one case, SC8’s (40) comprehensive support structure and varied activities facilitated growing engagement for a group of Syrian refugee families who had spent between 2–4 years in the country. In this intervention, participants are described as having progressed from passive participation to active ownership and design of the project, with the garden evolving into a safe space for help-seeking, where complex political, social, and cultural problems could be discussed. The provision of interpretation and support with Danish language learning (e.g., shed space used for vocabulary lists in Arabic and Danish for vegetables and tools) and open policy for using the space in leisure time enabled participants to develop social capital through space ownership, growing peer connections, and in time through bridging relationships with allotment neighbors and authorities. The intervention’s success in fostering empowerment manifested through participants’ increasing autonomy and an evolved relationship with municipal authorities as collaborators, with the space being integrated into the municipal integration programme for case work meetings and events.

Conversely, SC24’s (71) outcomes illustrate the challenges arising when profound unmet support needs exist and minimal enabler provision is made, even when participants are deemed to be “settled” or “established” refugees. The intervention was designed for refugees who were currently unemployed but had started an education programme at a Danish language school, who were assessed as not ready for work due to health issues without having a formal diagnosis. Of an initial 37 participants, 9 left the programme for diverse reasons, including severe mental illness, pain, or having started a job. In this study, participants expressed that they lacked the ability to balance challenges and cope with them, due to loss of identity through forced migration. Ongoing trauma was described as an important barrier to having the energy or power for personal growth and to establish in a new country context. Participants also notably felt under pressure from authorities’ expectations, with some describing feeling unsafe in society and feeling unwilling to go outside. Although participants who completed the scheme reported positive experiences through both gardening and positive contact with the villagers, reflections noted that the learning process can be hindered by feelings of loss of identity, acculturative stress and traumatic experience. Creating a safe and secure environment, opportunities for positive contact with others, being met with respect, trusted with meaningful activities and learning new skills were reflected as critical for education or employment initiatives with a similar group.

These contrasting outcomes demonstrate that the right enablers function as more than facilitation: they are critical to addressing systemic issues rather than surface symptoms. SC8’s (40) comprehensive support created conditions for community-building through negotiation and coproduction that supported individual and community empowerment. In comparison, for the participants that completed the programme, SC24’s (71) participants gained some confidence and skills and saw the experience as positive, but still noted ongoing unaddressed needs and lack of confidence in managing ongoing challenges in an atmosphere of alienation from authorities. This evidence suggests that insufficient support provision may not only limit engagement but may inadvertently reinforce marginalization by highlighting deficits without the structures and/or pathways to address these issues. This also challenges assumptions that residence duration alone determines support needs, as the impact of traumatic experience, systemic barriers, and acculturative stress persist regardless of time spent in country, and require recognition to enable genuine participation rather than mere attendance.

### Synthesis of evidence gaps and research priorities

Identifying evidence gaps is integral to answering our research questions in a realist review: understanding what works, for whom, and in what circumstances requires equal attention to where evidence is insufficient to inform confident recommendations. Five programme theories were assessed as “weak” or “very weak” (minimal empirical evidence, primarily theoretical, representing research needs for validation) based on available evidence, identifying critical research priorities validated through expert advisory consultation.

#### Trusted referral pathways support higher engagement

##### Evidence strength: very weak|evidence: cross-cutting|social prescribing phase: access

Despite theoretical and practical importance to social prescribing uptake and system flow, evidence regarding referral pathways into interventions is largely absent. Recruitment processes were clearly described in only 21 of 34 included study clusters, with little comparative data on referral effectiveness. Examples include SCs 22, 26, and 29 (46, 47, 72) for brief comparative data across referral pathways. Some indications are given of the value of trusted recommendations as, e.g., SC26 (46) describes warm handovers where participants had heard about the programme from the bicultural worker, friends or family, SC22 (72) describes a large proportion of self-referral through awareness raiding in an Initial Accommodation Centre. This is then contrasted in SC29 (47) where sporadic and low numbers for referrals from health/social services are discussed (although this was the source of most referrals compared to community partners). However, systematic assessment of the importance of trusted referral pathways is not possible based on evidence reported. Research priorities include controlled comparisons of referral pathways and longitudinal monitoring of engagement trajectories based on referral sources to better understand engagement and uptake.

#### Matching migration stage and needs supports better outcomes

##### Evidence strength: weak|evidence: cross-cutting|social prescribing phase: access

Timing for intervention participation and migration stage-appropriate matching was largely unreported, an area with clear theoretical importance for social prescribing access and delivery. Time since arrival was inconsistently reported across studies with specific settlement duration documented only in 8 study clusters (and then with huge variation), legal status also only in 8 (typically not in detail). Similarly, intersectionality and multiple marginalization factors were not discussed in detail but suggested by other population characteristics in 12 interventions, and a needs-based assessment was included in 12 interventions (although this was typically not further described). It was stressed in conversation with the expert advisory board that migration stories are not linear and support must be responsive to lived realities, which are often outside of individual control. Without consistent reporting of migration stage and needs or longitudinal tracking of changing needs over time, understanding optimal matching remains speculative. Research priorities include prospective cohort studies tracking refugee needs across settlement phases, comparative effectiveness studies of interventions delivered at different migration phases, and development of validated assessment tools for determining migration phase timing and readiness to engage with different support types.

#### Community bridging supports mainstream service integration

##### Evidence strength: very weak|evidence: cross-cutting|social prescribing phase: access

Evidence regarding referral pathways “back” from community settings into mainstream health care or social prescribing is limited but would be useful to understand. System linkage and sustainability of interventions through system integration were rarely reported, with most studies focusing on intervention delivery as “one-off” models. Examples where further system integration was achieved were often centred in or alongside institutional settings, such as SC26 (46) focusing on maternity services for refugee groups where changes were integrated into multiple hospital sites, or SC8 (40) as a community garden initiative, which was initiated alongside a municipal integration programme and became integrated as an event and case worker space. No studies indicated long-term service utilization patterns post-intervention or compared outcomes between those accessing services directly versus through community bridging. The absence of evidence is particularly concerning given policy emphasis on avoiding parallel service systems, whereby referral back to mainstream service is likely practiced and supported by community organisations but this is simply not visible in published research. Research priorities therefore include longitudinal studies tracking service utilization trajectories from community to mainstream services, comparative effectiveness research on bridged versus direct access (linked to the referral research priority above), and identification of critical bridging functions that facilitate successful transition.

#### Acute crisis impacts uptake and participation

##### Evidence strength: weak|evidence: cross-cutting|social prescribing phase: access

Crisis thresholds and their impact on intervention appropriateness remain largely unreported, with weak evidence based primarily on exclusion criteria rather than empirical testing. Several studies excluded individuals in acute crisis, sometimes without defining thresholds or providing alternative pathways. Examples include SC16 (73) which excluded refugees with any contagious illness, physical or psychological condition that might disrupt work at the community centre, whereas SC3 (59, 60) described providing alternative referrals for families in crisis who were then not enrolled in the intervention. This absence in the data prevents us from forming an understanding of when social prescribing and/or community referral is inappropriate or counterproductive. It is also challenging as refugee individuals in acute crisis may well still present in social prescribing settings, even though this is not systemically designed to be the responding service. In conversation with the expert board, this research priority elicited mixed responses based on practical realities: although all felt that non-crisis services or community organisations were not appropriate responders, these may be the only available support and in individual cases may be a needed lifeline through a crisis. Further work is needed to understand how best to proceed with a systemic approach, as acute crisis is known to be relevant for this population, crisis services remain constrained in their capacities, and not all subgroups can access all crisis services (e.g., undocumented migrants).

#### Intersectional marginalization impacts access and quality of care

##### Evidence strength: very weak|evidence: cross-cutting|social prescribing phase: access

Within-refugee population diversity remains largely unreported and unexamined in the evidence based, with only 8 study clusters explicitly addressing intersectional disadvantage for multiply-marginalized subgroups. Particularly clear examples in this regard include SC22 (72), which defines and illustrates examples of multiple disadvantage that may impact young people with forced migration experience, or the “canvas ceiling” example given by SC14 (65) focusing on individual-level and structural factors impacting employability for women who have experienced domestic violence. Disability status, physical health status, LGBTQIA+ identity, and religious minority status (among other relevant characteristics) go unreported across the evidence base. This absence of disaggregated outcome data impacts understanding of how subgroups facing even greater barriers access, engage, and can sustain continued participation in services or community. This is critically relevant to social prescribing and understanding differential effects, which is particularly concerning given the likelihood that standardized models may systematically exclude the most marginalized.

## Discussion

### Summary of key findings

This rapid realist review sought to assess evidence on social prescribing for refugee populations (RQ1) and to generate insights into the same from social-capital based interventions (RQ2). Our findings demonstrate that while formal social prescribing evidence for refugee populations is limited, insights can be derived from social-capital based interventions which are operationalized in comparable ways. The majority of included evidence therefore addresses RQ2, providing insights into what approaches may work, for whom, and under what circumstances when applied to social prescribing contexts.

We identified five intervention families representing distinct operational approaches: Comprehensive barrier-reduction models (providing intensive multi-domain support with extensive practical enablers); co-produced navigation models (emphasizing cultural brokerage through lived experience); trauma-responsive therapeutic models (structured, peer-led trauma processing in safer spaces); Community-connected social capital models (relationship-building in trusted environments); and minimal support skills-training models (targeted skill development for established populations). These families represent primary operational modes that characterize how interventions are delivered and how participants engage, and are not mutually exclusive. Especially examples of comprehensive barrier-reduction models may integrate one or multiple other family models in their service delivery in real-world settings.

Drawing from this evidence base and after prioritization by our stakeholder group based on relevance to social prescribing, 15 programme theories were defined. These were organized around key dimensions of social prescribing delivery: accessing social prescribing (referral pathways); in social prescribing appointments (initial uptake and population-specific needs); and onward referral from social prescribing (supporting ongoing participation). Five statements were coded as strong to moderate-strong based on available evidence: co-produced service navigation uncovers hidden barriers; trauma work requires safer spaces and appropriate professional support; building new relationships requires safer social surroundings; childcare provision promotes attendance and engagement; and barrier-support matching supports attendance and engagement. Five statements were coded as weak or very weak based on available evidence, aligned with accessing social prescribing, which are discussed in detail under research priorities below.

### Interpretation of key findings

Intervention appropriateness and effectiveness for refugee populations depends fundamentally on alignment between contextual barriers, activated mechanisms, and available support infrastructure. Comprehensive barrier-reduction models are most appropriate for multiply-marginalized populations experiencing multiple barriers or intersecting disadvantages, for instance for recent arrivals, caregivers, those in poverty and conflict survivors. In these contexts, extensive practical enablers (e.g., childcare, food, interpretation, transport) activate engagement mechanisms that would otherwise remain dormant, enabling the possibility of effectiveness. Conversely, minimal support skills-training models are only appropriate for “established” or “settled” refugee persons who have existing capacity and agency, where targeted skill development may be effective without intensive wraparound support. This variation underscores that migration stage, stability, individual needs, and pre-existing capacity or agency (including how transferable or relevant these are in the new country context) are likely critical moderators determining which intervention approaches can meaningfully engage different refugee subgroups or individuals.

Several cross-cutting concepts emerged through data synthesis as particularly relevant for inferred insights for social prescribing. Structured session plans were often coded alongside affiliated broader features for trauma-informed practice: peer leadership, cultural adaptation, community-led staffing, and unstructured social time. Fittingly to principles of trauma-informed practice (74–76), all these features and enablers are indicated as contributors to creating the psychological and relational safety necessary for trauma processing. Our analysis suggests that co-production similarly operated beyond formal participatory structures—it appeared to function as a bidirectional knowledge exchange mechanism where refugees’ experiential expertise revealed system barriers not visible to professionals, while simultaneously building individual navigation capacity. We conceptualized safety as both spatial (trusted community locations) and relational (through peer witnessing and experiential validation), enabling individuals to open to disclosure and building connections. These concepts indicate that effective and appropriate social prescribing services for refugee populations likely require simultaneous attention to multiple reinforcing elements, particularly seen in delivery culture and interpersonal skills, rather than singular “tick-list” components.

The evidence highlighted migration stage, marginalization, crisis and readiness dynamics as critical yet under-researched and little-understood contextual factors. Migration stage may moderate support intensity needs and capacity to engage with different intervention types. For instance, trauma work likely requires baseline emotional and social stability, whereas navigation support may be acutely needed early on but remain valuable across phases as individuals need to engage with systems that are new to them. Multiple marginalization profiles compound barriers in ways that single-axis interventions likely fail to address: refugee mothers, torture survivors, and unaccompanied youth require approaches that recognize intersecting barriers. Resource infrastructure requirements varied substantially by intervention family and focus, with evidence suggesting that insufficient enablers may not only limit engagement but potential reinforce marginalization by highlighting deficits without providing structures where these can be addressed. Evidence suggested likely gaps in system provision for those in acute crisis, as alternative pathways to crisis services may not be equitably available and community settings may remain the only accessible support for individuals (even if not matching their delivery focus or capacity to support).

However, the reliance on social-capital intervention evidence to inform social prescribing practice requires caution in future implementation. Mechanisms which are observed in peer-led, community-embedded and often co-produced interventions are here assumed to be replicable within formal social prescribing structures. In reality, these may lack equivalent relational infrastructures, community embeddedness, and workforce capacity. Social-capital interventions were frequently delivered by facilitators who shared lived experience or who had built trust over time in community settings, and with flexible intensity and duration which is rarely accommodated in formal commissioning frameworks. Importing only the surface features of the model without the wider structural conditions that activate their underlying mechanisms risks producing superficially similar delivery environments with missing mechanistic activations. This is particularly concerning for multiply-marginalized refugee populations, where the difference between a well-resourced, relationally safe intervention and an under-resourced approximation likely determines whether engagement is possible at all.

### Contribution to literature

This review makes several distinct contributions to the social prescribing evidence base. First, it provides the first systematic synthesis of evidence specifically addressing refugee populations, addressing a critical gap in a literature base dominated by dominant populations in high-income settings. By demonstrating that formal social prescribing evidence for refugees is exceptionally limited but that substantial insights can be derived from aligned social-capital based interventions, we establish both the current evidence landscape and propose a methodological approach for leveraging lessons from related intervention research. The intervention family typology offers a conceptual framework for understanding operational diversity in refugee-focused community services. This conceptualisation identifies linked mechanisms and outcomes that shape appropriateness and effectiveness and may be a useful aid to social prescribers considering how to support refugee individuals.

Second, this review extends social prescribing theory by articulating CMO patterns specific to refugee populations, particularly around co-production as bidirectional knowledge exchange, safety in spatial and relational elements, and enabler intensity as a matching requirement. Third, our findings connect social prescribing, social-capital based interventions, and forced migration literature in novel ways. We demonstrate that social capital building for refugees operates through distinct mechanisms, including experiential validation, cultural brokerage, and spatial safety, that likely differ from mechanisms central to privileged dominant population evidence, where assumptions of legal status, stable housing, and system familiarity rarely require such direct attention. Social prescribing could represent a potentially valuable approach for greater refugee health equity, provided that implementation attends to specific individual contextual needs and can support appropriate referrals within capacity and resourcing for the wider service and community landscape.

### Strengths and limitations

There are several strengths to this review, including its broad search strategy combining conventional and tailored approaches with extensive outreach to capture grey literature. Evidence provided by individual charity organisations in the form of service reports is important and central to the final findings. It is also a limitation of this review that further service reports were not returned through the tailored approach, likely due to capacity in the community sector and a lack of written evidence. This practice-based evidence would have been particularly valuable to real-world implementation analysis. In order to not miss out on important insights available in verbal form, an accompanying expert interview series was conducted (37). This also ensured that members of the review team, who were also involved in expert interview data analysis, benefitted through data coding and synthesis from these wider stakeholder perspectives and priorities. This further stakeholder engagement continued in the form of wider discussions with members of the expert advisory board for this review, who provide perspectives spanning academic, practitioner, statutory and community practices and were an invaluable support to final programme theory prioritization and wordings.

Equally, this review has several limitations. There was insufficient evidence to comprehensively answer RQ1. Furthermore, the final study clusters represented exceptionally diverse interventions, with limited longitudinal data, small sample sizes, and substantial heterogeneity in reporting, population characteristics, features, and outcomes. The evidence base’s geographic concentration in minority-world contexts, particularly the United States, Australia and European countries, limits transferability to majority-world settings where most refugees reside. Population reporting is a major limitation for this review, as legal status, time since arrival, and distinct ethnic and cultural characteristics often went unreported or were reported imprecisely, restricting context-specific analysis. Further categories of migrants who often are missing from systemic perspectives, specifically undocumented migrants and economic migrants, were largely absent from the evidence base, as was evidence in reporting of marginalized subgroups (e.g., LGBTQ+ refugees, elder refugees, refugees with physical health conditions or with disabilities, religious or ethnic minorities). This limits insights into appropriateness and effectiveness for those facing compounded barriers who comprise some of the most disadvantaged groups in our society. These evidence gaps mean programme theories should be understood as preliminary inferences requiring further validation and attention from research and practice, and implementation in diverse contexts should proceed with accompanying evaluation frameworks to test local applicability.

### Research priorities

We identify five research priorities. First, systematic assessment of referral pathway effectiveness through controlled comparisons of trusted community referrals, institutional referrals, and self-referral, also considering the role of supported referrals, with longitudinal monitoring of engagement trajectories. Second, prospective cohort studies are needed to identify and assess refugee needs through migration stages, with comparative effectiveness studies delivered in different migration stages, and development of validated assessment tools for determining migration stage, timing and appropriateness for referrals, and readiness to engage with different support models. Third, longitudinal studies tracking service access and utilization for referrals back to mainstream health or social care services from community are needed, including comparative effectiveness research on bridging versus direct access pathways, and identification of critical bridging functions that support successful transition. Fourth, empirical investigation of crisis thresholds for refugee populations and the impact on social prescribing appropriateness, including developing clear crisis identification protocols for social prescribers where these do not exist and alternative pathways models where no other accessible support exists. Fifth, disaggregated access and outcome data is needed to understand trajectories for refugee individuals accessing social prescribing, including for those who experience intersectional marginalization. All studies should prioritize reporting of migration stage in terms of legal status, time since arrival, and key demographics for future meta-analyses and systematic evaluation.

### Policy and practice recommendations

Before outlining recommendations, it is important to acknowledge the structural conditions under which these must be implemented. Social prescribing workforce capacity and work scope is already stretched (77). The additional competencies required for refugee-responsive practice represent substantial training and resourcing requirements that are not currently funded or mandated in most commissioning frameworks. Practical enable provision (e.g., particularly interpretation, childcare, transport, food) is rarely built into social prescribing or third sector commissioned budgets. However, the evidence reviewed here suggests that these are not optional add-ons but mechanistically necessary conditions for engagement. Commissioning cycles that prioritize short-term outputs and throughput or “service user flow” metrics are likely structurally misaligned with the kind of sustained adaptive, relationship-dependent support this population requires (77). These should not be seen as arguments against implementation. These should be seen as arguments for honest and appropriate resourcing. The recommendations below should therefore be understood as contingent on funding and commissioning environments being developed to match the complexity of the task, rather than as achievable purely within current standard social prescribing frameworks.

Health systems and commissioners must recognize that social prescribing for refugee populations requires distinct implementation approaches and consideration, rather than assuming models designed for dominant populations can be extended without adaptation. A service that is designed to be responsive to individual needs with infrastructure to support marginalized refugee persons is likely to be better placed to respond to all individuals experiencing multiple barriers. Commissioning frameworks should also accommodate practical enabler provision as core components when serving multiply-marginalized populations. Investment in bicultural, multilingual social prescribing workforce development would support cultural brokerage. Referral pathway development should prioritize sustainable trusted community partnerships as well as institutional pathways, with resources allocated for relationship-building and exchange with refugee-serving organisations as partners. Integration with existing refugee support services requires genuine co-design with refugee communities and organisations rather than parallel system development, including clear sustainable pathways for referrals between statutory and community settings. These efforts should be institutional and systemic instead of being dependent on key personnel, as in event of staff turnover these connections may not be recovered and mapping of local assets may not exist in a centralized form that can then be accessed by others. Critically, commissioners and policymakers should address the tension between crisis exclusions and refugee lived realities—although social prescribing is not a crisis response and is not systemically positioned to pick up such cases, it is likely that this occurs. This situation requires likely both crisis response capacity development and robust alternative pathway creation, in collaboration with community partners who equally experience the same tensions. Finally, monitoring and evaluation frameworks should mandate disaggregated data collection on individuals accessing social prescribing, including refugee subgroups, their migration stages, and onward trajectories to enable evidence accumulation on what works, for whom, and under what circumstances for these populations from social prescribing experiences directly.

## Conclusion

This rapid realist review demonstrates that while formal social prescribing evidence for refugee populations remains limited, substantial insights can be derived from social-capital based interventions which were operationalized in comparable ways. Nevertheless, concerning research and evidence gaps exist which are deserving of attention from policy, research and practice communities.

We suggest that with the proliferation of social prescribing programmes internationally, investment and policy attention should be harnessed within high-quality concomitant research and evaluation frameworks that also specifically focus on marginalized migrated populations. Such programmes should therefore mandate disaggregated data reporting, including migration status, baseline characteristics, acceptability and reach for social prescribing, experience with onward referrals, and longer-term individual trajectories. The evidence examined here indicates the level of complexity and specialist knowledge needed to build a truly responsive service with greater equity in delivery, where services in general need both practical tools in appointments and strong links to wider community services to better address refugee individuals’ needs with holistic, barrier-responsive support. Simple signposting at initial presentation for multiply-marginalized refugee persons is unlikely to be sufficient without considering intensive wraparound supports that address practical, relational, and structural needs simultaneously. Considering situations where small or rural localities see a sudden influx of asylum seekers due to distribution policies, it is easy to see how existing social prescribers and services will not be equipped to immediately respond without further evidence-based support, training, protocols, and strong collaboration with local assets from community leaders to refugee-serving organisations. The insights from this review raise fundamental considerations for the training, welfare and support of social prescribers, social prescribing service culture in diverse settings, interpersonal approaches in social prescribing appointments, and resource and capacity allocation.

Critically, even with deep understandings of refugee individual needs and robust links between health and community services, social prescribing is unlikely to represent a cure-all solution. As our intervention family typology demonstrates, appropriateness and effectiveness depend fundamentally on alignment between contextual barriers, activated mechanisms, and available support infrastructures. This will always be individual-dependent, where what works for refugee persons with relevant existing capacity and agency bears little resemblance to what multiply-marginalized recent arrivals may need. Equally, particularly in current policy climates, the question of when a refugee person is “settled” or “established” cannot be answered categorically or universally and long-term accompaniment is likely critical to individual growth. The non-linear nature of various unpredictable crises in settlement, including for status change, health, trauma recovery, and experienced discrimination, means that it is misguided to impose blanket timeframes on improving individual wellbeing. This represents a systemic tension for social prescribing models, if short-term accompaniment or signposting delivery forms dominate with matching metrics on service flow representing the only valued data. Further systemic tensions remain unresolved, particularly considering crisis exclusions and pathways in contexts where social prescribing may be the only accessible support for refugee persons, event when not designed or resourced as such.

Social prescribing has potential to advance refugee health equity, but only if implementation actively recognizes the need for distinct approaches in delivery, adequate resourcing (also applicable to community partners), sustained investment in workforce development and welfare, and committed collaboration with refugee communities and refugee-serving organisations. Appropriateness and effectiveness will remain dependent on complex interactions between individual refugee needs and circumstances, migration contexts, resource availability, and relationships across health-community landscapes, rather than on any standardized model.

## Supplementary material

SUPPLEMENTARY DATA SHEET 1 Search strings for conventional approach.

SUPPLEMENTARY DATA SHEET 2 Rapid review process summary table.

SUPPLEMENTARY DATA SHEET 3 Theory-data evidence mapping summary.

SUPPLEMENTARY DATA SHEET 4 Expert board deck.

SUPPLEMENTARY TABLE 1 Full lists of literature at each screening stage.

SUPPLEMENTARY TABLE 2 Extraction table.

SUPPLEMENTARY TABLE 3 Data synthesis file.

SUPPLEMENTARY TABLE 4 Included studies.
