# Supplementary material for: Social prescribing for refugee populations: a rapid realist review of international evidence
Source: Front Public Health. 2026 May 8;14:1758335. doi: 10.3389/fpubh.2026.1758335 (PMC13194373; doi:10.3389/fpubh.2026.1758335)
Supplement: SUPPLEMENTARY DATA SHEET 3 — Theory-data evidence mapping summary. [file Data_Sheet_3.pdf]

Theory-Data evidence mapping summary

Document Purpose

This document maps evidence from 34 study clusters to 23 programme theory statements. For each statement, we provide:

- The theoretical claim
- Supporting evidence
- Type of evidence (pattern, mechanism, inference)
- Confidence rating

How to interpret evidence

- Strong: Clear patterns across multiple studies, good mechanism evidence
- Moderate: Observable patterns but mechanisms inferred; or strong patterns with limited mechanism evidence
- Weak: Minimal evidence, primarily theoretical, identifies research need for validation

Statement distribution

By evidence strength:

- Strong: 8 statements
- Moderate: 10 statements
- Weak: 6 statements

By category:

- Cross-cutting (Sx): 7 statements
- Family-specific (Fx): 5 statements
- Contextual (Cx): 4 statements
- Additional (Ax): 7 statements

Statements with strong or moderate evidence

Statement 1 (S1): Burden-Intensity Matching Principle

Confidence: Moderate-Strong

Theory statement: Support intensity (enabler provision) must align with population barrier burden. Highly disadvantaged populations are more likely to require comprehensive support (4-8 enablers); stabilized populations require selective support (2-4 enablers); established populations experiencing lower barriers can access with minimal support (1-3 enablers).

Supporting evidence:

Pattern evidence (strong):

- Family 1 (n = 10): Mean 5.5 enablers (range 4-8), serving refugee families with children, recently resettled, women survivors of domestic violence, urban refugees in precarious situations, multiply-marginalised groups (Study IDs: 3, 4, 6, 8, 20, 22, 23, 25, 31, 34)
- Families 2-4 (n = 18): Mean 2.3-2.8 enablers (range 1-4), serving mixed populations, some established groups, meeting specific needs (navigation, trauma management, social connection) (Study IDs 1, 2, 5, 9, 10, 11, 12, 15, 17, 18, 21, 26, 27, 29, 30, 32, 33)
- Family 5 (n = 7): Mean 2.1 enablers (range 1-3), serving mixed adult groups, legal status mostly not-specified (Study IDs: 7, 13, 14, 16, 19, 24, 28).
    - Some arguable outliers: serving established refugees but who are unable to work without a medical diagnosis (ID 24); serving established women who are survivors of domestic violence (ID 14)

Mechanism evidence (moderate – inferred):

- ID 23 (8 enablers): Comprehensive support enabling participation for highly disadvantaged young people (e.g. SEN)
- ID 3 (5 enablers): Link worker model with comprehensive support for families with mental health needs
- ID 31 (7 enablers): Exercise programme for refugee women with higher enabler provision to enable family participation

Types of evidence:

- Cross-sectional pattern (populations served cluster with enabler provision)
- Intervention design choices (designers matched support to populations served)
- No experimental manipulation or testing evidence

Evidence gaps:

- No studies tracking what happens when vulnerable populations access low-enabler interventions
- No longitudinal population burden assessments linked to enablers
- Matching is implied from design, not tested experimentally

Statement 2 (S2): Focus-Feature Alignment Principle

Confidence: Strong

Theory statement: Intervention characteristics match intervention goals through mechanistic requirements e.g. navigation associates with the feature co-production (cultural brokerage mechanism); trauma recovery associates with trauma-informed and peer-led features (safer structures and shared witness mechanisms); social capital associates with trusted locations (spatial safety mechanism); barrier reduction associates with comprehensive enablers (accessibility mechanisms).

Supporting evidence:

Navigation & co-production (strong):

- Family 2: 86% co-produced (6 of 7 navigation interventions)
  - Co-produced: IDs 2, 5, 26, 29, 32, 33
  - Not co-produced: ID 15 (outlier) – but this is a mentoring intervention, so argue it is a reasonable assumption to assume some degree of co-production even when not explicitly stated in text and then extracted

Trauma recovery & trauma-informed approaches / peer-led approaches (strong):

- Family 3: 80% trauma-informed (4 of 5), 60% peer-led (3 of 5), 40% community-led staff (2 of 5)
  - Trauma-informed: IDs 1, 10, 18, 27
  - Peer-led: 10, 12, 18
  - Community-led staff: 10, 18

Social capital & trusted location (strong):

- Family 4: 60% trusted location (3 of 5)
  - Trusted location: 9, 11, 30

Barrier reduction & comprehensive enablers (strong):

- Family 1: Defining family characteristic (4+ enablers)

Types of evidence:

- Cross-sectional feature or enabler distribution
- Intervention design patterns
- No experimental tests of feature/enabler removal

Evidence gaps:

- No studies testing e.g. navigation without co-production versus with co-production
- Limited negative case analysis (ID 15 arguably co-produced in reality through mentoring but not explicitly stated)
- Mechanisms described but not always empirically demonstrated

Statement 3 (S3): Resource-Type Prioritization Principle

Confidence: Moderate

Theory statement: Different interventions require different resource mixes – material (childcare, transport, food), knowledge (system information, cultural brokerage), relational (social connection, therapeutic relationships), or structural (trusted locations, social time). Resource types aren't freely substitutable for intervention families.

Supporting evidence patterns by family:

- Family 1 (material primary): Childcare 80%, Transport 40%, Food 60%, Equipment 50%
- Family 2 (knowledge primary): Co-production 86%
- Family 3 (relational primary): Trauma-informed 80%, Peer-led 60%, Community-led staff 40%
- Family 5 (minimal further resourcing): Referral to further support 57%

Resource mix evidence suggests that families achieve effectiveness with different enabler types (not quantities).

Types of evidence:

- Descriptive pattern of resource allocation
- Variation in feature/enabler types across families with similar counts
- No direct tests of resource substitution

Evidence gaps:

- No studies testing whether resource types are substitutable
- Resource type effectiveness not directly measured
- Synthesis interpretation rather than explicit study findings – only comparison case is between IDs 33 and 34, where the second is a better-resourced extension of the first study cluster

Statement 4 (S4): Co-production Domain Specificity

Confidence: Moderate

Theory statement: Co-production and peer leadership effects are domain-specific, strongest for navigation (86% in Family 2) and trauma recovery (60% peer-led in Family 3), where lived experience provides unique expertise. For other intervention types, professional facilitation may be sufficient.

Supporting evidence:

- Family 2 (Navigation): 86% co-produced
  - IDs: 2, 5, 26, 29, 32, 33 (15 arguably not an outlier)
  - Mechanisms activated: Cultural brokerage, refugee system navigation expertise
- Family 3 (Trauma): 60% peer-led, often then in combination with community-led staff (66%)
  - IDs: 10,12, 18
  - Mechanism: Shared witness, experiential authority
- Other families have lower co-production or peer-led patterns, not a defining feature

Types of evidence:

- Distribution patterns across families
- Theoretical alignment of feature with mechanisms

- No direct within-domain comparisons

Evidence gaps:

- No explicit tests of co-production value for different family or intervention types
- Peer-led intervention effectiveness not compared to professional-led

Statement 5 (S5): Equity-Efficiency Trade-Off

Confidence: Moderate

Theory statement: Services cannot simultaneously maximise equity (serving most marginalized and multiply-marginalized groups) and efficiency (cost per participant). Comprehensive support (Family 1) costs more per capita but reaches populations minimal support excludes. Minimal support (Family 5) maximises scalability but systematically excludes highly marginalized populations.

Enabler provision gradient:

- Family 1 (Barrier reduction) 5.5 – highest resource intensity;
- Family 3 (Trauma) 2.8;
- Family 4 (Social capital) 2.4;
- Family 2 (Navigation) 2.3;
- Family 5 (Skills) 2.1 – lowest resource intensity

Population served comparison:

- Family 1: Multiply-marginalized, recently resettled, families with children, survivors of domestic violence
- Family 5: Mixed adult groups, legal status mostly not-specified

Types of evidence:

- Descriptive patterns of resource allocation and populations served
- Inverse relationship between enabler provision and scalability (theoretical inference)
- No economic evaluation data or exclusion measurements

Evidence gaps:

- No cost-per-participant or cost-per-outcome data
- No systematic assessment of who’s excluded by which models (drop-out rates also not systematically recorded)
- Trade-off inferred from resource levels, not empirically demonstrated

Statement 6 (S6): Enabler Stratification Philosophy

Confidence: Moderate

Theory statement: Enabler provision stratification (5.5 → 2.8 → 2.4 → 2.3 → 2.1) reflects fundamentally different design philosophies about accessibility, not quality variation. Family

1 designed to remove all barriers, Families 2-4 to match strategic enablers or features to needs, Family 5 assumes existing capacity.

Supporting evidence:

Stratification pattern and enabler/feature-focus matching (strong): Clear gradient and clustering across families

Philosophy evidence (moderate – inferred):

- Family 1: Diverse enabler types to enable multiply-marginalized to participate (Childcare 80%, Transport 40%, Food 60%, Equipment 50%);
- Families 2-4: Strategic prioritization
- Family 5: Assumes existing capacity, focus on referrals outwards for further support (57%)

Types of evidence:

- Patterns and clustering across enablers and features
- Intervention design choices and populations served
- Philosophy is an interpretive framework for patterns

Evidence gaps:

- Designer rationales not explicitly stated across interventions
- Alternative explanations possible (funding constraints vs. intentional design)
- Proposed philosophy is synthesis interpretation

Statement 7 (S7): Feature-Focus Synergies

Confidence: Strong

Theory statement: Certain features create synergies with specific foci through mechanism linkage. For instance, co-production amplifies navigation potential, trauma-informed approaches amplify trauma recovery potential, trusted locations amplify social capital potential. These are synergistic, representing necessary conditions, and not just additive.

Synergy 1: Co-production & Navigation (strong)

- 86% co-produced (6 of 7 navigation interventions)

Synergy 2: Trauma-informed & Trauma recovery (strong)

- 80% trauma-informed (4 of 5), 60% peer-led (3 of 5), 40% community-led staff (2 of 5)

Synergy 3: Trusted location & Social capital (strong)

- 60% trusted location (3 of 5)

Types of evidence:

- Feature clustering with specific foci

- Near-universal inclusion suggests necessity → co-production, trauma-informed approaches
- No experimental tests of feature or enabler removal

Evidence gaps:

- No studies testing synergy versus necessity distinction
- Clustering doesn't prove multiplicative effects
- Alternative explanation: well-designed interventions include appropriate features/enablers

Statement 8 (F1): Enabler Density Creates Accessibility Equity

Confidence: Strong

Theory statement: Comprehensive barrier reduction is essential for engaging multiply-marginalized refugees. Below a resourcing threshold (~4 enablers), multiply-marginalized groups may not be able to access services regardless of their quality.

Supporting evidence:

- Family 1 characteristics (strong); key example IDs include 8, 20, 23, 31, 34
- Population distribution across families, as families 2-5 with <4 enablers serve different populations
- Threshold inferred from clustering, not tested

Mechanism evidence:

- Barrier reduction to enable attendance and participation
- Dignity affirmation as resource investment communicates worth
- Capacity enablement and increased agency

Types of evidence:

- Consistent design pattern
- Population-enabler alignment
- No experimental threshold testing

Evidence gaps:

- No direct comparison of vulnerable populations with varying enabler levels
- Threshold of 4 is observation, also created through family allocation decision-making, not a tested cut-off
- Some borderline cases (ID 6 at 4)

Statement 9 (F2): Navigation Requires Refugee Experiential Expertise

Confidence: Strong

Theory statement: System navigation interventions require co-production with refugees, because professionals lack experiential knowledge of barriers, cultural considerations, and effective strategies to overcome these.

Supporting evidence:

- Family 2 characteristics (strong): 86% (6 of 7, ID 15 arguably not true outlier)

Mechanism evidence:

- Cultural brokerage as refugees understand cultural barriers professionals may miss
- Experiential knowledge derived from lived experience navigating systems
- Trust and credibility when refugees are matched with peer navigators
- Language and cultural matching act as supports

Types of evidence:

- Strong clustering pattern (86%), single negative case (ID 15) which is argued to not be a true outlier, also without comparative data
- Mechanism descriptions and inferences from interventions

Evidence gaps:

- No experimental tests of co-production necessity
- No evidence to assert causation rather than correlation

Statement 10 (F3): Trauma Work Requires Safety Architecture and Peer Connection

Confidence: Strong

Theory statement: Trauma-responsive interventions require trauma-informed approaches creating structural safety and setting expectations, peer leadership providing shared witness and experiential authority, and cultural adaptation to ensure resonance.

Supporting evidence:

- Family 3 characteristics (strong): mean 2.8 enablers
  - Trauma-informed: 80%
  - Peer-led: 60%
  - Community-led staff: 40%
  - Culturally adapted: 80%
- ID 10 as key example of full feature integration
- Feature synergy between trauma-informed, peer-led, culturally-adapted, and community-led staff (% given from sub-group that have at least one feature to indicate how many have both), e.g.
  - Trauma-informed & peer-led (50%)
  - Trauma-informed & culturally-adapted (75%)

    - Peer-led & community-led staff (66%)

Mechanism evidence:

- Safety architecture: trauma-informed approach using same session structure sets expectations and creates greater psychological safety to participate
- Shared witness: peers with trauma experience provide validation
- Experiential authority: peer facilitators have credibility from lived experience
- Cultural resonance: culturally adapted approaches ensure relevance

Types of evidence:

- Feature clustering in trauma-focused interventions
- Multiple features co-occurring
- No studies detailing trauma interventions without these features

Evidence gaps:

- No comparison of trauma interventions with trauma-informed approaches
- Trauma-informed approach restricted to a single characteristic (same structure) due to observed clustering of that feature – other trauma-informed approaches noted in other interventions but inconsistently described, often with missing detail
- No peer-led versus professional-led effectiveness comparisons
- Features cluster but their necessity is not experimentally demonstrated

Statement 11 (F4): Social Capital Benefits From Trusted Location Infrastructure

Confidence: Strong

Theory statement: Social capital building interventions benefit from trusted locations and social exchange as foundational infrastructure creating spatial safety that legitimizes relationship formation.

Supporting evidence:

- Family 4 characteristics (strong)
    - Trusted location (60%)
    - Unstructured social time (40%)
    - Referrals for further support (40%)
    - Group-based as primary delivery mode (60%)

Mechanism evidence:

- Spatial safety: physical spaces where refugees feel safe taking social risks
- Relationship legitimization: trusted setting validates new relationships, opportunity for bonding outside of set programmes

- Community infrastructures: Community hubs enabling repeated contact, often culturally appropriate locations

Types of evidence:

- Structural/relational enabler clustering (trusted location / unstructured social time / referrals for further support)
- Mechanism descriptions

Evidence gaps:

- No comparison of trusted location versus in clinical settings for group-based work focusing on social connection
- Trusted location included private household spaces as well as community spaces, difficult to differentiate between impact of interventions held in household spaces versus community centres

Statement 12 (F5): Skills Transfer Assumes Existing Access Capacity

Confidence: moderate

Theory statement: Minimal-support skills interventions (Family 5) assume participants already possess independent access capacity. Low enabler provision (range 1-3, mean 2.1) reflects either appropriate resourcing for established populations or under-resourcing through other constraints that creates exclusion issues.

Supporting evidence:

- Family 5 characteristics (strong): Mean 2.1 enablers, lowest across families, no Family 5 intervention with 4+ enablers, all are skill-based (focused on employability = 3; health literacy = 2; activity = 1; psychosocial skills = 1)
- Enabler distribution
    - 1 enabler: 2 IDs 13, 19
    - 2 enablers: 2 IDs 16, 24
    - 3 enablers: 3 IDs 7, 14, 28
- Assumed capacity evidence is inferred
    - Interpretation: 1/7 (14%) versus 9/10 in Family 1 (90%)
    - Childcare: 2/7 (29%) versus 8/10 in Family 1 (80%)
    - Transport: 2/7 (29%) versus 4/10 in Family 1 (40%)
    - Food: 2/7 (29%) versus 6/10 in Family 1 (60%)

Two possible interpretation frameworks:

- Interpretation A: Appropriate resourcing
    - Engaging refugees who can access independently (contraindicated by individual IDs e.g. 14, 24)
    - Efficient design matches low-barrier population needs
    - Skills content is the focus, not barrier reduction
- Interpretation B: Under-resourcing creates exclusion potential

    - Low enablers exclude populations who need skills (perhaps supported through drop-out rates reported e.g. IDs 7 or observations that intervention inadequate to meet needs e.g. ID 13)

Evidence cannot distinguish between interpretations:

- Individual ID reporting of relatively high drop-out rates (ID 7), or drop out before intervention started (ID 24), moderate effects without significant change for some variables assessed (ID 28), or observations of intervention inadequacy to address need (ID 13)
- Populations not consistently described – majority of interventions did not describe legal status of populations
- No available data on exclusion due to barriers or dropout due to unaddressed barriers

Types of evidence:

- Enabler provision levels and types
- Skills focus areas
- Assumed capacity inferred from missing enablers
- Populations descriptions are limited

Evidence gaps:

- Cannot determine if appropriate targeting or under-resourcing
- No comparison of established vs. recent refugees in this family
- No consistent exclusion data

Statement 14 (C2): Geographic and Resource Context Adaptation

Confidence: Moderate

Theory statement: Evidence base is predominantly from high-income, urban, Global North contexts. Interventions may require substantial adaptation for low-resource, rural, or Global South contexts.

Supporting evidence:

Geographic distribution (strong):

- High-income countries: majority representation at 91%
    - USA most often represented with 13 interventions – 38% of all study clusters
- Upper middle-income countries: South Africa 3% (ID 20)
- Lower middle-income countries: Lebanon 6% (IDs 7 and 27)
- Low-income countries: no representation

Setting distribution:

- Urban: Often not explicitly stated, but based on setting descriptions majority assumed where not otherwise described (e.g. camp or rural setting) 30 IDs at 88% of all study clusters
- Refugee camp setting: 1 ID (ID 27) at 3% of all study clusters
- At least partial rural delivery: 3 IDs (ID 8, 15, 23) 9% of all study clusters

Adaptation requirements (theorized):

- Enabler types and investment differ (urban transport ≠ rural transport)
- Trusted locations or important cultural community centres may not exist (need alternative infrastructure)
- Co-production may require more capacity building
- Comprehensive support may be cost-prohibitive – IDs range from 1 enabler (27, refugee camp) to 8 enablers (23, described as also covering rural settings through presumed predominantly urban), difficult to compare how much was rural delivery for IDs 8 and 23, compared to 15 which is explicitly set in a rural town

Types of evidence:

- Geographic descriptions
- No studies comparing same intervention across differing country or setting (urban/rural) contexts
- No explicit adaptation studies
- Difficult to compare studies that were partly delivered in rural settings

Evidence gaps:

- No low-income country comparison studies
- No rural versus urban comparison studies
- No cost comparisons across contexts or settings
- Adaptation requirements are theorized only

Note: This is primarily an evidence gap statement. The gap itself is well-documented and was explicitly addressed as likely in the review's protocol. Adaptation requirements are only theorized.

Statement 15 (C3): Organizational Capacity Requirements

Confidence: Moderate

Theory statement: Different families require different organizational capacities e.g. Family 1: substantial funding, logistics coordination, partnerships; Family 2: co-production infrastructure, refugee employment; Family 3: trauma-informed culture, supervision; Family 4: trusted spaces, community relationships and exchange; Family 5: minimal infrastructure, linkage for onward referrals.

Supporting evidence (inferred from intervention design):

- Family 1: funding for high enablers provision, logistics coordination for multiple enablers, multi-agency partnerships with close collaboration crucial (childcare, transport, interpretation, referrals)
- Family 2: co-production infrastructure and refugee employment inferred to require appropriate compensation, training, power-sharing processes, and cultural humility within the organizational culture
- Family 3: trauma-informed culture, with high peer-led and community-led staff representation inferred to require appropriate training and clinical supervision for facilitators
- Family 4: Trusted physical spaces, unstructured social time and referrals for further support inferred to require good community relationships and cultural legitimacy, appropriate management for group activities
- Family 5: Minimal enabler infrastructure but high representation of referral into other services inferred to require good partnership linkage

Observed delivery mode alignment:

- Link worker → case management capacities
- Hybrid models → multi-component coordination, multi-agency partnership work
- Group-base → space and facilitation needs
- Structured programmes → curriculum delivery
- Peer-led → recruitment, training, support systems

Types of evidence:

- Inferred from intervention requirements (also logical necessity, for instance in case of high enabler provision)
- Minimal reporting of cost per capita or savings per capita (IDs 22, 23)
- No explicit organizational capacity assessments
- No implementation or failure studies due to capacity gaps

Evidence gaps:

- Organizational capacity not directly measured
- Partnership structures generally under-described (particularly in terms of allocated roles, responsibilities, funding)
- Costs not reported
- Staffing models inconsistently detailed
- No studies of capacity-related failures

Note: Synthesis of what interventions logically require, not empirical capacity assessment.

Statement 16 (C4): Gender and Family Structure Considerations

Confidence: Strong

Theory statement: Childcare provision is especially critical for interventions targeting women. Family-oriented interventions may require different enabler packages than individual-focused.

Supporting evidence:

- Childcare provision concentrates in the comprehensive support model (Family 1)
    - Family 1 – 8 of 10 (80%)
    - Family 2 – 1 of 7 (14%)
    - Family 3 – 1 of 5 (20%)
    - Family 4 – 0 of 5 (0%)
    - Family 5 – 2 of 7 (29%)
- Women-focused interventions are more likely to provide childcare when part of the comprehensive barrier reduction model (ID examples 20, 31)
- Family oriented (IDs 3, 8, 17) often provided childcare (66%)
- Further interventions worked primarily with children or young people, sometimes involving their wider families (IDs 4, 7, 12, 13, 22, 23) with lower provision of childcare (33%)
- Gender separated spaces offered or designed: 8 of 34, 24%, (IDs 4, 7, 10, 11, 21, 25, 27, 28)
- Explicitly women only groups: 9 of 34, 26% (IDs 4, 11, 14, 20, 26, 27, 29, 31, 32); predominantly women groups 3 of 34, 9% (IDs 3, 6, 33)

Types of evidence:

- Childcare provision rates
- Gender focus of interventions
- Participant genders
- Gender separated groups

Evidence gaps:

- Single parent subgroup and family gender roles not consistently visible in data
- Men's participation barriers and caregiving responsibilities under-studied and discussed
- Intersections with further characteristics not assessed

Statement 17 (A1): Enabler Combination and Threshold Effects

Confidence: Moderate

Theory statement: If enabler provision reaches threshold for family type (e.g. 4 for comprehensive barrier reduction model), then core mechanisms likely activate. Beyond that threshold, incremental benefits may occur.

Supporting evidence

- Enabler ranges within families and distribution e.g.

    - Family 1 (barrier reduction): 4 - 30% (IDs 4, 6, 25); 5-6 - 50% (IDs 3, 8, 20, 22, 34); 7-8 - 20% (IDs 23, 31)
    - Family 2 (navigation): 1-2 – 71% (IDs 2, 5, 15, 26, 33); 3 – 14% (ID 32); 4 – 14% (ID 29)
    - Family 3 (trauma): 1-2 – 40% (IDs 18, 27); 3 – 20% (ID 1); 4 – 40% (IDs 10, 12)
    - Family 4 (social capital): 1-2 – 60% (IDs 9, 17, 30); 3 – 20% (ID 21); 4 – 20% (ID 11)
    - Family 5 (skills): 1-2 – 58% (IDs 13, 19, 16, 24); 3 – 43% (IDs 7, 14, 28)
- Threshold evidence (moderate): also influenced by family allocation choices, boundaries suggested but unclear whether functional threshold or designed
- Combination effects (weak – untested): outcome data not systematically comparable or linked to enablers, cannot isolate from other variables

Theoretical considerations:

- Diminishing returns – are the first enablers critical up to 4 (or in key combinations depending on intervention focus), following enablers are incremental, comprehensive support not always needed because fewer barriers remain?
- Linear benefit – does each additional enabler add value to participants engagement and attendance by removing barriers?
- Cannot distinguish with available data

Types of evidence:

- Range data (descriptive)
- Clustering patterns
- Only one case example of same intervention with additional enablers 33 → 34, but arguably context and setting differences confound evidence for additional enabler benefit and this is also not a focus of outcome discussion (large time difference between, different populations in different towns, different delivery structures) → Ergo, no controlled comparisons

Evidence gaps:

- No studies testing "3 vs. 4 vs. 5" enablers with same or comparable populations in comparable settings
- Threshold is observed clustering, partly informed by family allocation design choices, not tested cut-off
- Optimal enabler combination for family focuses not able to be assessed

Statement 19 (A3): Peer Leadership Scope and Support

Confidence: Moderate

Theory statement: If peer leaders are provided with training, supervision, compensation, and clear roles, then they can activate cultural brokerage and shared witness mechanisms while maintaining wellbeing. Without support infrastructure, peer burnout and role confusion undermine effectiveness and outcomes.

Supporting evidence:

- Peer leadership distribution (moderate): Family 1 (barrier reduction), Family 2 (navigation), Family 3 (trauma), Family 5 (skills)
- Peer roles observed:
    - Family 1 (barrier reduction): Trained refugee facilitators (ID 3); Peer leadership in defining intervention goals and supporting each other with STEM work (ID 4); Trained community members as health educators (ID 6); Peers decided garden design and delivery (ID 8); Community refugee women as peers (ID 20); Peer mentor roles (ID 22); Trained peer facilitators and professional helpers (ID 25)
    - Family 2 (navigation): Refugee-background community liaison workers
    - Family 3 (trauma): Bicultural staff and peer facilitators (ID 10); Graduates became mentors for new recruits (ID 12); Mentors were refugee volunteers (ID 18)
    - Family 5 (skills): Paraprofessional peer facilitators from the community (ID 28)
- Support infrastructure (moderate): inconsistently reported, some interventions include great detail on training, supervision, and compensation (e.g. IDs 3, 25), others provide very little detail (IDs 12, 22)
    - Specific mention of difficulties created by unclear role boundaries reported in outcomes for one study (ID 5)

Mechanism evidence:

- Cultural brokerage through shared background
- Experiential authority through lived experience
- Shared witness impact for greater validation
- Trust and credibility through peer connection#

Inferred support needs (theoretical):

- Training: systems knowledge, cultural brokerage, trauma-informed approaches, health literacy, boundaries
- Supervision: case discussion, problem-solving, secondary trauma processing
- Role clarity: scope of support, when and where to refer, professional backup for trauma contexts, education not clinical advice

Types of evidence:

- Peer involvement rates and types (quantitative/descriptive)
- Peer role descriptions

- Support infrastructure for staff training and roles (where reported)
- Mechanisms described

Evidence gaps:

- Peer training not consistently described
- Supervision not consistently described and content or purpose often not reported
- Compensation typically not reported
- Peer wellbeing often not reported or assessed
- No supported versus unsupported peer comparisons
- No peer-led failure studies

Note: Peer-led elements well-documented, exact content and support infrastructures often unclear. Stakeholder validation needed.

Statements requiring further research and stakeholder validation

Statement 13 (C1): Temporal Positioning and Readiness

Confidence: Weak – requires validation

Theory statement: Interventions show different optimal timing in refugee settlement phases: posited that Families 1 (barrier reduction) or 2 (navigation) are most effective early during stabilization, Family 3 (trauma) requires stabilization, Family 5 (skills) assumes establishment and readiness for growth; Family 4 (social capital) could work across the timeline.

Supporting evidence:

- Temporal data (weak): most studies do not report time since arrival
    - Occasionally picked up under population description as recently arrived or resettled, but not typically qualified

Inferred temporal patterns:

- Family 1 (burden reduction) – early. Population descriptions suggest recent arrival, high barrier burden typical of crisis phases.
- Family 2 (navigation) – early to mid. Navigation needs typically acute when systems unfamiliar and may change over time.
- Family 3 (trauma) – mid. Theorized as trauma and mental health work requires some stability and is contraindicated in cases of acute crisis (where crisis management is then advised).
- Family 4 (social capital) – flexible after initial settlement. Forming social relationships requires some stability but could occur across the timeline.

- Family 5 (skills) – established. Assumed existing capacity and readiness for growth or advancement.

Theorized sequencing only – trajectories are likely to also vary by population group depending on language skills, cultural barriers, and professional and personal backgrounds, so should not be assumed relevant to all refugee individuals.

Individual person-centred readiness assessment needed.

Type of evidence:

- Mostly theoretical/logical inference
- Population descriptions occasionally suggest timing with some indication of gradient
- No systematic temporal data
- No longitudinal studies

Evidence gaps:

- Time since arrival rarely reported
- No longitudinal tracking
- No timing comparison studies
- Individual variation by needs assessment not captured
- Population variations not captured
- Readiness assessment or needs-based assessments not described when captured

Stakeholder input needed as this is primarily a theoretical framework.

Identified research need for prioritization.

Statement 18 (A2): Referral Pathways and Engagement

Confidence: Very weak – requires validation

Theory statement: If refugees are referred from trusted persons (community organisations, peer networks, culturally-matched providers), then initial engagement is higher due to legitimacy transfer. Warm referrals with cultural brokerage activate trust mechanisms that cold referrals do not.

Supporting evidence:

- Referral pathway data (very weak):
  - Referral sources not systematically reported in most studies
  - Descriptions focus on content and delivery, not referral intake pathways
- Limited evidence:
  - Some interventions were supported by recruitment within community centres or via community organisations (IDs 4, 5, 6, 11, 22, 25, 30)
  - Self-referral versus formal referral rarely detailed (IDs where this is assumed possible 4, 5, 6, 17, stated for IDs 22, 23, 31)

    - Peer/word of mouth mentioned occasionally but not consistently reported (ID 26)

Mechanism evidence (theorized):

- Legitimacy transfer: Trusted person's recommendation transfers trust to intervention (no specific data captured to corroborate, closest in ID 26)
- Cultural brokerage in referral supports engagement (no specific data captured, examples could include pathways as with ID 16 where participants were recruited from a community event)
- Trust acceleration through warm referral (no specific data captured, can be inferred from partnerships described in ID 22)

Types of evidence:

- Minimal available from referral pathways descriptions after extraction
- Theoretical framework based on logical inference and delivery locations or cultural adaptation
- No empirical data on referral sources or effects

Evidence gaps:

- Referral sources often not reported, nearly no description of individual roles in referral ("warm referrals")
- Referral process inconsistently described
- No comparison of referral pathway effects on attendance or engagement
- Self-referral versus formal referral rarely mentioned and cannot be analysed from available data
- Warm versus cold referral not tested
- Trust transfer not measured

Stakeholder input needed as this is primarily a research gap statement based on matching missing data to the social prescribing referral model.

Identified research need for prioritization.

Statement 20 (A4): Responsive Implementation and Learning

Confidence: Very weak – requires validation

Theory statement: If organizations implement interventions with monitoring systems tracking engagement, barriers, outcomes and have capacity to adapt based on data, then interventions optimize for population / context. Rigid implementation without monitoring may allow mismatches to persist.

Supporting evidence:

- Monitoring and adaptation data (very weak):

    - Implementation adaptation processes largely not described in detail that indicates monitoring or context changes led to a change in delivery (exceptions: IDs 10, 22, 23, 26)
    - Studies report fixed outcomes, not adaptation processes
- Evaluation evidence (limited):
    - Studies broadly report outcomes (cross-sectional, post measures, pre-post measures, occasionally pre-, mid-, and post- data collection points)
    - Monitoring during implementation with focus on delivery process not described
    - Changes between IDs 33 and 34 infer changes to implementation based on first experiences e.g. greater range of enablers and for specific delivery focuses including English proficiency, social support, and re-establishment of valued social roles
- Adaptation evidence (limited):
    - How interventions were adjusted based on early feedback or experience: largely not reported (Exception: ID 23 – e.g. changed referral path focus, employed culturally matched staff)
    - Whether enablers were added or removed during delivery: largely not reported (Exception: ID 23 – e.g. exchange translation via devices to personal translators)
    - How timing or content was modified: largely not reported (Exceptions include IDs 10 and 26 due to COVID-19)
    - Organisational learning is captured across multiple studies (IDs 2, 6, 10, 13, 22, 29, 31, 32, 33) including identifying facilitator training as essential, process improvements or adaptive processes.

Mechanisms (theorized):

- Monitoring enables adaptation: tracking barriers reveals what is missing or needed for engagement
- Dropout patterns shows mismatches (very little reported data)
- Participant feedback guides modifications (very little reported data)
- Rigid implementation fails to address context specifics

Types of evidence:

- Theoretical from logical inference and implementation science
- May have happened but is largely invisible in final research reporting – broadly, it is more visible in included service evaluations (e.g. IDs 22, 23) than published articles, which may also explain this data hole

Evidence gaps:

- Implementation adjustments largely not described
- Monitoring systems for process adjustments largely not reported
- Adaptation mechanisms not detailed

- Organisational learning not consistently reported
- Rigid versus flexible service delivery not compared

Stakeholder input needed as this is primarily a research gap statement based on matching missing data to implementation science principles.

Identified research need for prioritization.

Statement 21 (A5): Mainstream Service Integration

Confidence: Weak – requires validation

Theory statement: If refugee-specific interventions function as bridges to mainstream services (building capacity, cultural navigation, warm handoffs), then refugees develop sustainable access beyond the intervention. If interventions or services create parallel systems without mainstream service integration, then time-limited dependency is created without long-term integration pathways.

Supporting evidence:

- Mainstream integration data (weak):
    - Connections to mainstream statutory services are rarely explicitly described (Exception: IDs 6, 8, 17, 19, 22, 23, 26)
    - Integration into mainstream services after time-limited interventions broadly not reported (Exception: IDs 8, 15, 26)
    - Studies broadly focus on intervention delivery, not systems integration
    - Most appear essentially refugee-serving within community, which may then represent parallel systems (but unclear as not reported)
    - Warm handoffs not described, transition planning rarely mentioned (Exceptions as above, IDs 8, 15, 26), post-intervention pathways largely unclear

Mechanisms (theorized):

- Bridge model: Intensive support initially → builds capacity and connections → facilitates transition → sustained mainstream access
- Parallel systems model: Refugee services separate → refugees access during intervention or engagement with service → services end, lack mainstream alternatives → dependency created on time-limited services

Types of evidence:

- Connections to mainstream services or integration after delivery end rarely described
- Referrals in or out of service occasionally described but not in detail that indicates whether warm referrals took place

- Integration outcomes not measured for how the service becomes a part of existing systems
- Parallel versus bridge model assumptions cannot be assessed

Evidence gaps:

- Mainstream connections are not described
- Post-intervention and transition pathways not tracked in terms of continued access to services
- Integration versus parallel systems cannot be assessed
- Bridge model effectiveness cannot be assessed
- Sustainability of most included studies unknown
- Handoff processes not detailed

Stakeholder input needed as this is primarily a research gap statement based on matching missing data to a critical systems question for access.

Identified research need for prioritization.

Statement 22 (A6): Failure Conditions and Contraindications

Confidence: Weak – requires validation

Theory statement: If populations are in acute crisis (homelessness, extreme poverty, imminent deportation, acute mental health crisis), then even comprehensive support may be insufficient for engagement as survival imperatives override.

If organizations lack essential infrastructure for family type (e.g. Family 2 navigation without co-production), then implementation may fail.

Supporting evidence:

- Failure condition data (very weak):
    - Essentially not reported, studies report successes not failures
    - Two studies indicate referring participants in acute crisis onwards or not allowing enrolment based on acute crisis or other diagnosis (e.g. ID 3, 16)
    - Implementation challenges related to crisis rarely described (Exception describing dropout before intervention start: ID 24)
    - Dropout reasons not systematically analysed (dropout rarely reported – exception ID 7)
    - Publication bias toward success

Contraindication evidence (theorized):

- Acute crisis threshold prevents engagement (e.g. homelessness, imminent deportation, mental health crisis requiring stabilization through clinical care)

- Family 1 (barrier reduction): acute crisis may overwhelm even comprehensive support
- Family 2 (navigation): without co-production capacity, intervention may fail
- Family 3 (trauma): without trauma-informed capacity, intervention risks retraumatization or inability to accompany individuals appropriately
- Family 4 (social capital): without structural /relational supports such as trusted location, unstructured social time, referrals onwards, intervention may fail to create safer space
- Family 5 (skills): without readiness matching, highly marginalized populations may be excluded

Types of evidence:

- Mostly absent (no populations in acute crisis, no failed studies)
- Inferred from missing data with theoretical reasoning about mechanism failure

Evidence gaps:

- No crisis threshold testing
- No implementation failure studies
- No contraindication testing
- Very limited dropout data reported and not broadly analysed
- Population exclusion reasons broadly not reported

Stakeholder input needed as this is primarily a research gap statement based on logical inference with matched missing data.

Identified research need for prioritization.

Statement 23 (A7): Intersectional Marginalization Response

Confidence: Very weak – requires validation

Theory statement: If interventions explicitly design for multiply-marginalized subgroups (refugee women with disabilities; LGBTQIA+ refugees, elderly refugees, unaccompanied minors), then additional specialized adaptations required beyond standard service and enablers (e.g. accessibility accommodations, identity-safe spaces, age-appropriate approaches). If interventions assume refugee homogeneity, then most marginalized within refugee communities remain excluded.

Supporting evidence:

- Intersectional analysis (very weak, largely absent):

- Population descriptions typically very broad, with limited additional characteristics
    - Multiply-marginalized subgroups rarely identified and intersectionality rarely discussed (Exception: IDs 14, 22)
    - Within-refugee population diversity less often explored, when addressed this is often brief (IDs 11, 14, 15, 20, 22, 26, 27, 28, 29)
- Single axis identity focus seen for gender, age, family status in population definitions and intervention design
- Exceptions true for women survivors of domestic violence (ID 14) and unaccompanied refugee minors (IDs 22, 23 in part)
- Multiply-marginalised groups otherwise largely absent
    - Disabilities
    - LGBTQIA+
    - Elderly (exception IDs 33, 34)
    - Unaccompanied minors (exception IDs 22, 23)
    - Racial or ethnic minorities within refugee populations
    - Religious minorities
    - Special educational needs (exception ID 23)
- Within-group exclusion not measured
    - Who accessed versus excluded not disaggregated by intersecting identities
    - Dropout patterns not reported
    - Effectiveness variation based on intersectional identity not assessed

Mechanisms (theorized):

- Intersectional exclusion: standard interventions may exclude those with compounding marginalisation
- Wheelchair users or refugees with severe physical health conditions may not be able to access services despite other enablers
- LGBTQIA+ refugees may not be able to access community-based interventions if the community is hostile

Types of evidence:

- Minimal reporting of intersectional identities
- Intersectional analysis not possible
- Broadly no multiply-marginalized focus

Evidence gaps:

- Intersectional identities not identified
- Multiply-marginalised not reported

- Specialized adaptations broadly not reported (Exception: ID 23)
- Within-group diversity broadly not described (Exception: ID 23)
- Differential outcomes not measured
- Exclusion of multiply-marginalized not documented

Stakeholder input needed as this is primarily a research gap statement based on population characteristics assessment matched with missing data.

Identified research need for prioritization.

STATEMENT	EVIDENCE STRENGTH	KEY FINDING	ASSOCIATED STUDIES
**CROSS-SECTIONAL**			
**S1 Burden-intensity matching**	Moderate-Strong	Clear enabler-population patterns	1, 2, 3, 4, 5, 6, 7, 8, 9, 10, 11, 12, 13, 14, 15, 16, 17, 18, 19, 20, 21, 22, 23, 24, 25, 26, 27, 28, 29, 30, 31, 32, 33, 34
**S2 Focus-feature alignment**	Strong	Clear family focus-feature patterns	1, 2, 3, 4, 5, 6, 7, 8, 9, 10, 11, 12, 13, 14, 15, 16, 17, 18, 19, 20, 21, 22, 23, 24, 25, 26, 27, 28, 29, 30, 31, 32, 33, 34
**S3 Resource-type prioritization**	Moderate	Different families prioritize different resource types	1, 2, 3, 4, 5, 6, 7, 8, 9, 10, 11, 12, 13, 14, 15, 16, 17, 18, 19, 20, 21, 22, 23, 24, 25, 26, 27, 28, 29, 30, 31, 32, 33, 34
**S4 Co-production domain specificity**	Moderate	Co-production concentrates in F2 navigation and F3 trauma	2, 5, 10, 12, 15, 18, 26, 29, 32, 33
**S5 Equity-efficiency trade-off**	Moderate	Inverse relationship between enablers and scalability	1, 2, 3, 4, 5, 6, 7, 8, 9, 10, 11, 12, 13, 14, 15, 16, 17, 18, 19, 20, 21, 22, 23, 24, 25, 26, 27, 28, 29, 30, 31, 32, 33, 34
**S6 Enabler stratification philosophy**	Moderate	Enabler resourcing organised by design philosophies	1, 2, 3, 4, 5, 6, 7, 8, 9, 10, 11, 12, 13, 14, 15, 16, 17, 18, 19, 20, 21, 22, 23, 24, 25, 26, 27, 28, 29, 30, 31, 32, 33, 34
**S7 Feature-focus synergies**	Strong	Certain features cluster with specific foci	1, 2, 3, 4, 5, 6, 7, 8, 9, 10, 11, 12, 13, 14, 15, 16, 17, 18, 19, 20, 21, 22, 23, 24, 25, 26, 27, 28, 29, 30, 31, 32, 33, 34
**FAMILY**			

**F1 Enabler density creates accessibility equity**	Strong	Enabler density supports access and engagement for multiply-marginalised	3, 4, 6, 20, 22, 23, 24, 25, 31, 34
**F2 Navigation requires refugee experiential expertise**	Strong	Clear pattern of co-produced interventions	2, 5, 15, 26, 29, 32, 33
**F3 Trauma work requires safety architecture + peer connection**	Strong	Clear pattern of trauma-informed practice, peer-led and community staff features	1, 10, 12, 18, 27
**F4 Social capital requires trusted location infrastructure**	Strong	Social capital interventions are likely to take place in trusted locations	9, 11, 17, 21, 30
**F5 Transfer assumes existing capacity**	Moderate	Lowest enabler density, either as targeting or under-resourcing	7, 13, 14, 16, 19, 28
**CONTEXTUAL**			
**C1 Temporal positioning**	Weak	Research need: phased needs for settlement	Time since arrival rarely/inconsistently reported – inferred pattern theorized
**C2 Geographic adaptation**	Moderate	Evidence predominantly from Global North interventions	1, 2, 3, 4, 5, 6, 7, 8, 9, 10, 11, 12, 13, 14, 15, 16, 17, 18, 19, 20, 21, 22, 23, 24, 25, 26, 27, 28, 29, 30, 31, 32, 33, 34
**C3 Organizational capacity**	Moderate	Different families require different organisational infrastructure	1, 2, 3, 4, 5, 6, 7, 8, 9, 10, 11, 12, 13, 14, 15, 16, 17, 18, 19, 20, 21, 22, 23, 24, 25, 26, 27, 28, 29, 30, 31, 32, 33, 34
**C4 Gender/Family requirements**	Strong	Clear patterns for childcare provision; family vs individual differences	3, 4, 6, 7, 8, 11, 12, 13, 14, 17, 20, 22, 23, 25, 26, 27, 29, 31, 32, 33
**ADDITIONAL**			
**A1 Enabler combination**	Moderate	Clear clustering of enablers, combination effects unclear	1, 2, 3, 4, 5, 6, 7, 8, 9, 10, 11, 12, 13, 14, 15, 16, 17, 18, 19, 20, 21, 22, 23, 24, 25, 26, 27, 28, 29, 30, 31, 32, 33, 34
**A2 Referral pathways**	Very weak	Critical research need to understand importance of 'warm' referrals	Inward referral patterns nearly never reported
**A3 Peer support**	Moderate	Peer support roles require support infrastructure	3, 4, 5, 6, 8, 10, 12, 18, 20, 25, 28
**A4 Responsive implementation**	Very weak	Critical research need into service monitoring and adaptation to needs	Service monitoring and adaptation nearly never reported

**A5 Mainstream integration**	Weak	Research need into sustainable linkage beyond project lives	System linkage and sustainability rarely reported
**A6 Failure conditions**	Weak	Research need based on family inferences	Acute crisis needs inadequately reported
**A7 Intersectionality**	Very weak	Critical research need based on within-group diversity inferences	Intersectional considerations nearly never reported
