# Supplementary material for: Social prescribing for refugee populations: a rapid realist review of international evidence
Source: Front Public Health. 2026 May 8;14:1758335. doi: 10.3389/fpubh.2026.1758335 (PMC13194373; doi:10.3389/fpubh.2026.1758335)
Supplement: SUPPLEMENTARY DATA SHEET 4 — Expert board deck. [file Data_Sheet_4.pdf]

UNIVERSITÄT BIELEFELD

GRASP Uncertainties

Expert Advisory Board Deck

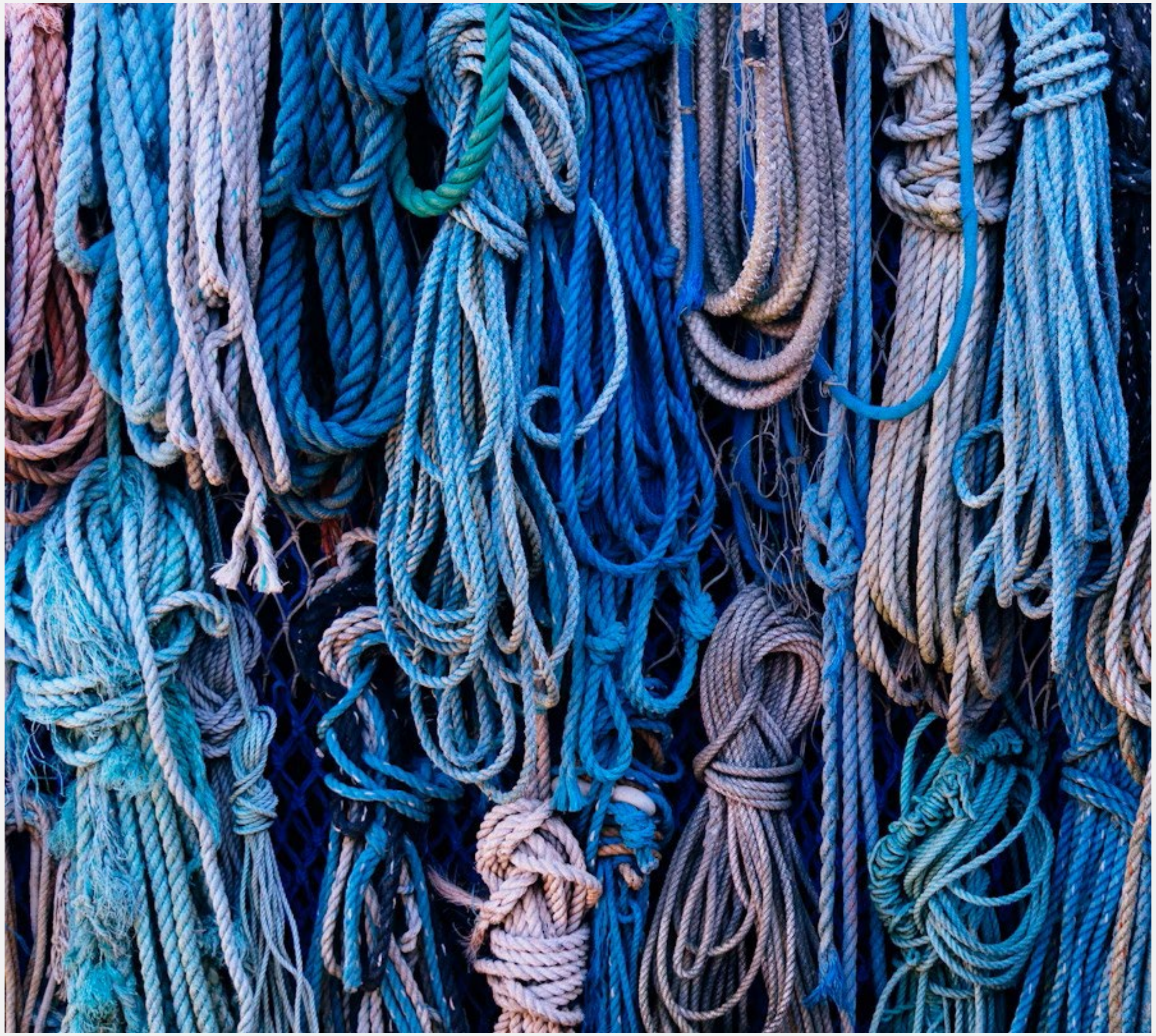

Victoria Touzel
GRASP Graduate School for “Health Systems and Policies in Uncertainties“
Public Health, University Bielefeld

UNIVERSITÄT BIELEFELD

GRASP Uncertainties

Slide deck outline

1. Review overview
2. Review findings for your feedback
    - 2.1 Introducing key terms and families
    - 2.2 Cross-cutting statements
    - 2.3 Family statements
    - 2.4 Contextual statements
    - 2.5 Additional statements

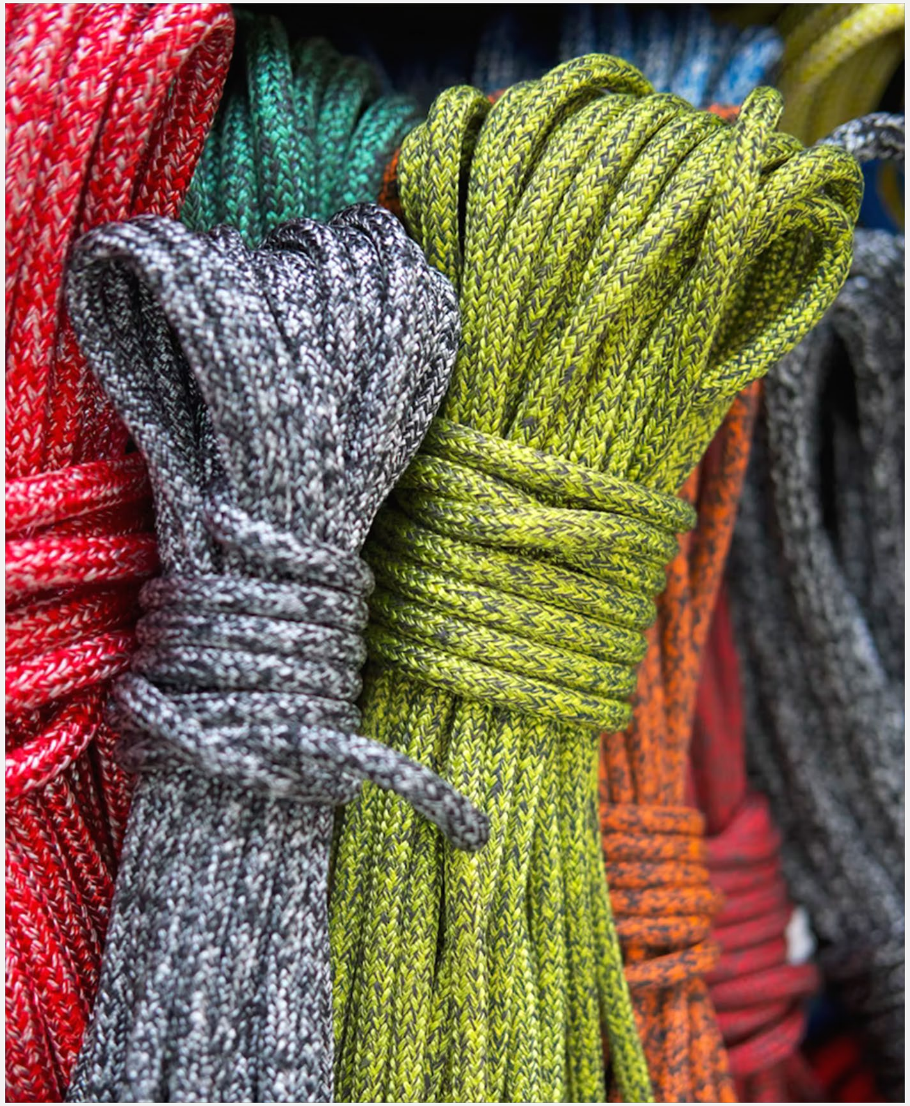

Reminder: Social Prescribing

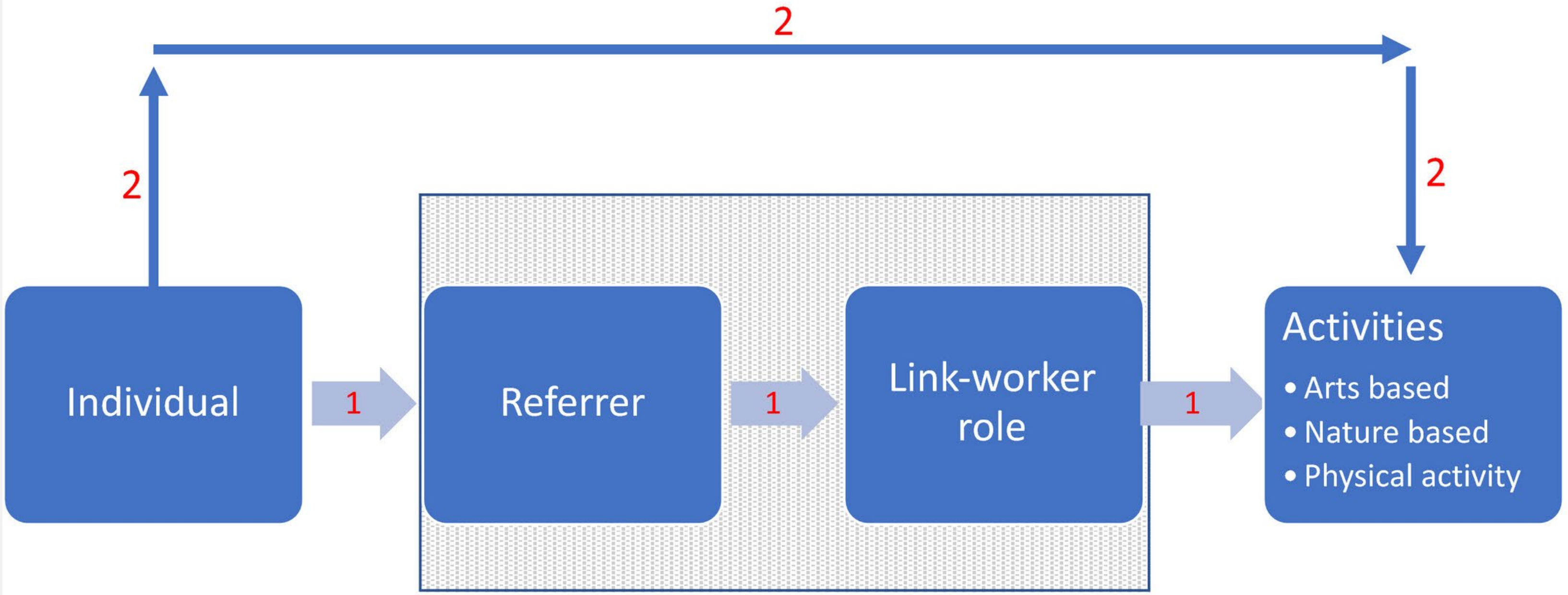

(1) – Social prescribing through primary care referral routes
(2) – Social prescribing through self-referral/community routes
Shaded box – health service 'scaffolding'

Hazeldine E, Gowan G, Wigglesworth R, Pollard J, Asthana S, Husk K. Link worker perspectives of early implementation of social prescribing: A 'Researcher-in-Residence' study. *Health Soc Care Community*. 2021; 29: 1844–1851. https://doi.org/10.1111/hsc.13295

1. Review overview

Realist review: Overview

Identifies relevant evidence from both social prescribing interventions, and social-capital based interventions (where comparable), considering evidence from RCTs, quantitative, qualitative, project reports or evaluations to answer:

RQ 1: What approaches to social prescribing focused on refugee populations work, for whom, and in what circumstances?

RQ 2: What additional insights can social capital-based interventions provide about which approaches with refugee populations may work, for whom, and in what circumstances?

Realist review: Timeline and strategy

Grey lit approach included:

- SP organisations and website searches
- Contacting relevant research centres and mailing lists
- Google, Google Scholar and Google Alert
- Additional database search (SOLO)
- Charity contacting from UK registers
- Citation chasing from included articles

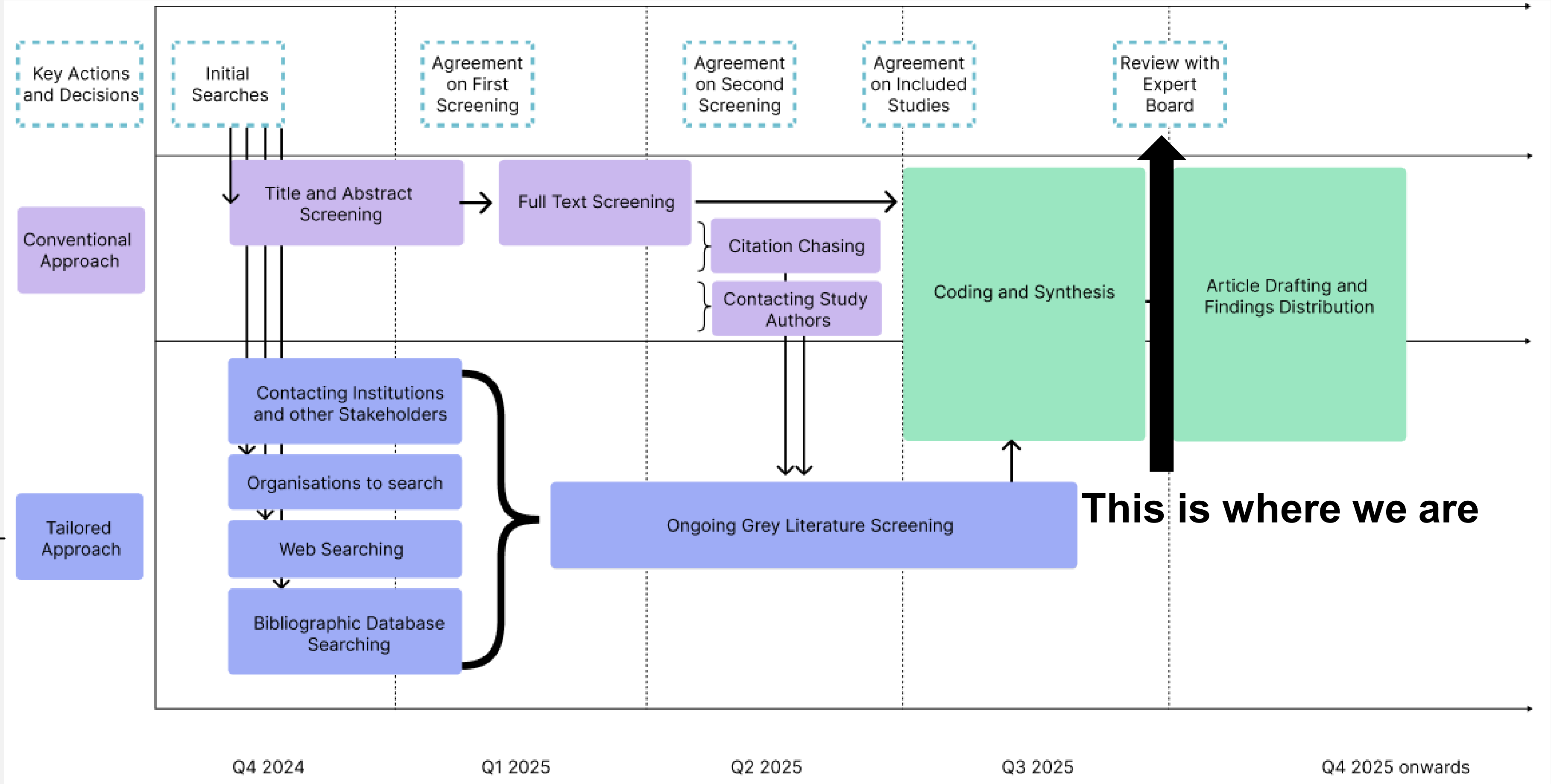

Realist review: Timeline and strategy

Source/Process step	Results	Date
Conventional	6,369	28.10.2024
Tailored (grey literature)	1,068	31.10.2024 – 21.02.2025
**Subtotal from searches:**	**6,369**	28.10.2024
Duplicates cleaned	2,486	28.10.2024
**Total in TiAb screening:**	**4,952**	21.02.2025
Excluded in TiAb screening	4,251	21.02.2025
**Total in full text screening:**	**701**	07.03.2025
Included from full text screening:	97	11.07.2025
Excluded after relevance, richness, rigour assessment:	58	11.08.2025
**Included in realist review for coding and synthesis:**	**39**	11.08.2025

UNIVERSITÄT BIELEFELD

2. Review findings for your feedback

Introducing key terms

- **Families:** Groups of interventions with shared goals and mechanism patterns (next slide)
- **Features:** The core design elements that enable mechanisms (or pathways for how an intervention works): Referral-led from health or social services, Peer-led, Culturally adapted, Skills-based, Co-produced, Trauma informed (same structures), Needs-based assessment
- **Enablers:** the supports that remove barriers *and* create the conditions for participation.
  - **Material/Practical:** food, transport, childcare, equipment, interpretation, community-led staff
  - **Structural/Relational:** trusted location, unstructured social time, referrals to further support

UNIVERSITÄT BIELEFELD

GRASP Uncertainties

Families identified from the evidence

Family name (Examples)	Family Definition	Primary Mechanisms	Core Outcomes
**Family 1: Comprehensive Barrier-Reduction Model** (IDs 23, 31, 34)	High support holistic models providing ≥4 enablers	Barrier removal → engagement, trust → relationship continuity, cultural safety → emotional safety, referral chaining → access expansion	Engagement continuity, access gain, moderate effect sizes but broad inclusion of marginalized groups
**Family 2: Co-produced Navigation Model** (IDs 5, 29, 32)	Co-designed and often peer-delivered interventions focused on navigating service systems	Co-production → accurate design, knowledge transfer → system literacy, empowerment → self-advocacy, peer mentors → trust in information	Improved service access, increased confidence and self-efficacy, reduced drop-off, emergent community advocates
**Family 3: Trauma-responsive Therapeutic Model** (IDs 1, 10, 12)	Mental-health-focused interventions, often community- or peer-led, and trauma-informed.	Trauma processing → regulation, psychological safety → engagement, cultural validation → sense of worth, peer modeling → hope activation	Reduced trauma and distress, improved coping and regulation, enhanced trust in services, greater participation, self-efficacy and hope
**Family 4: Community-Connected Social Capital Model** (IDs 9, 11, 21)	Group-based interventions using trusted spaces, social time, and cultural activities to build social capital.	Peer support → bonding capital, bridging capital → external networks, identity affirmation → belonging, hospitality → authentic connection	Reduced isolation, expanded networks and mutual aid, strengthened family/community ties, emergent collective identity
**Family 5: Minimal Support Skills-Training Model** (IDs 7, 14, 16)	Structured education or mentoring programmes which assume participants have access capacity	Knowledged and skills transfer → competence gain, Intrinsic motivation → learning engagement	Improved skills and knowledge, increased confidence for some but high dropout rate among those with unaddressed needs

Introducing key terms

- **Contexts:** The conditions or settings (such as stage of resettlement, gender, geography) that shape whether and how mechanisms are triggered.
- **Mechanisms:** The underlying processes that make programmes work, such as trust, cultural brokerage, shared witness, safety, or motivation.
- **Outcomes:** The changes or results produced when mechanisms work in context, such as engagement, attendance, trust-building, improved well-being, or integration.
- **Programme theory:** Explains how and why an intervention works — linking contexts, mechanisms, and outcomes (“If–Then–Resulting in” logic).

UNIVERSITÄT BIELEFELD

GRASP Uncertainties

If-Then statements

Evidence grade

IF [Context conditions]
THEN [Mechanisms activate]
RESULTING IN [Outcomes]

This means needs validation – your opinions are particularly important here to decide future research priorities

UNIVERSITÄT BIELEFELD

GRASP Uncertainties

C4. Gender and Family Structure Considerations

Strong

IF interventions engage women with children,

THEN childcare provision activates participation mechanisms by removing caregiving barriers,

RESULTING IN mothers' attendance and engagement; family-oriented interventions require different enablers than individual-focused.

We therefore infer that:

IF SPs engage with women with young children,

THEN connecting them with community organisations that provide childcare or spaces where children are welcome removes caregiving barriers,

RESULTING IN greater likelihood of mothers' attendance and engagement.

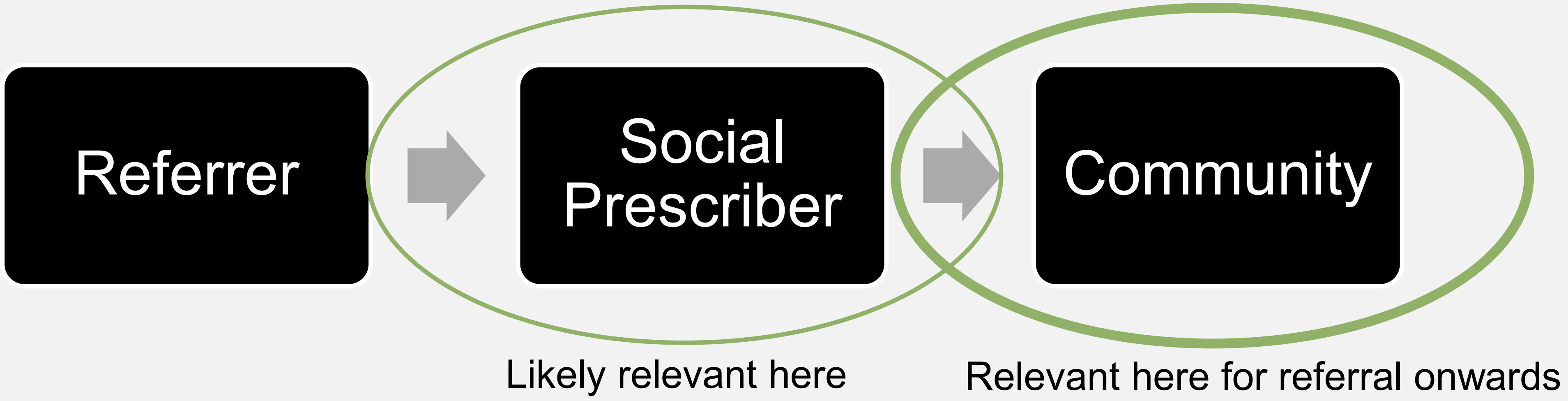

Feedback needed

1. **Clarity Check**: Does this statement make sense to you?

   -> Is there anything unclear or confusing about how it's worded?

2. **Experience Alignment**: Does this match what you've seen in practice?

   -> Where does your experience align with this, and where does it differ?

3. **Priority Rating:** How important is this statement on a scale of 1 – 5,

   where 1 is not important and 5 is very important?

C4. Gender and Family Structure Considerations

Strong

IF SPs engage with women with young children,

THEN connecting them with community organisations that provide childcare or spaces where children are welcome removes caregiving barriers,

RESULTING IN greater likelihood of mothers' attendance and engagement.

1: “Yes, makes sense" /
2: "Matches my experience with [specific example]" /
3: "Priority: 5 - this is essential”

UNIVERSITÄT BIELEFELD

Let’s get started!

Accessing Social Prescribing

A2. Referral Pathways and Engagement

Very weak

IF refugees are referred from trusted sources (community organizations, peer networks, culturally-matched providers) through warm referrals with cultural brokerage,

THEN legitimacy transfer activates trust mechanisms,

RESULTING IN higher initial engagement than cold referrals;
we cannot comment on self-referral from the evidence.

1. We therefore infer that:

IF refugees and asylum seekers are referred from trusted persons or organisations to SP or from SP to community organisations through supported referrals with appropriate accompaniment for that individual,

THEN that recommendation helps build trust,

RESULTING IN higher initial engagement than with unsupported referrals.

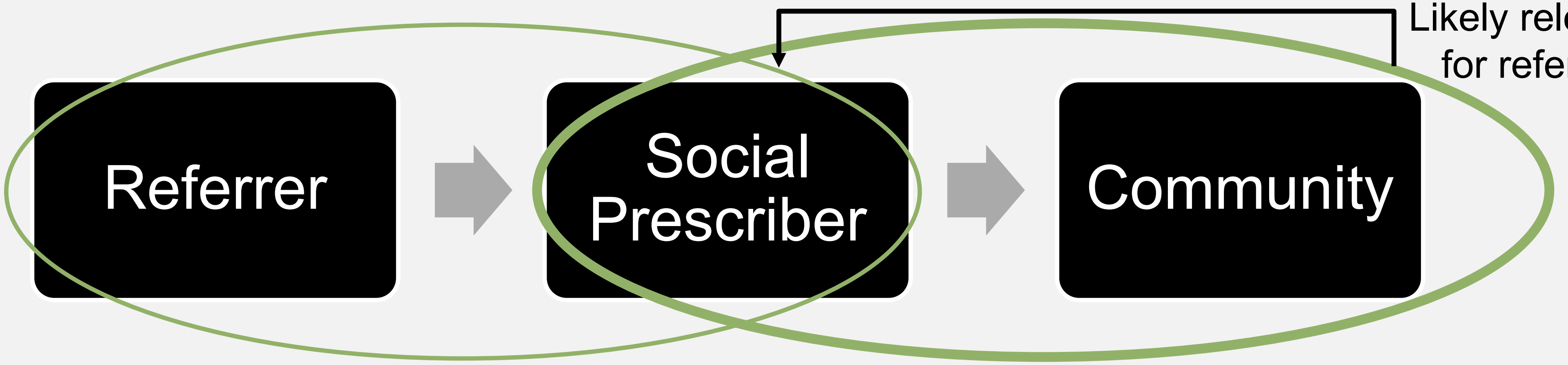

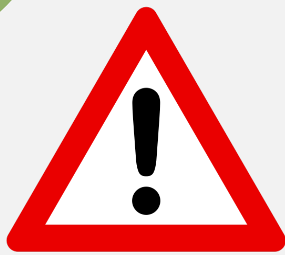

UNIVERSITÄT BIELEFELD

GRASP Uncertainties

C1. Temporal Positioning and Readiness

Weak

IF interventions are timed according to refugee acculturation phases (crisis -> settlement -> reestablishment) and/or existing capacity and skills,

THEN readiness-matched mechanisms activate (for instance, comprehensive support early, trauma processing mid, skills advancement later),

RESULTING IN optimal outcomes; mismatched timing reduces effectiveness; **we cannot comment on population differences from the evidence.**

2. We therefore infer that:

IF refugees and asylum seekers are referred to SP or from SP to community organisations based on their needs and stage of migration (e.g. newly arrived, socially/emotionally stabilized, settled),

THEN the referral aligns with the right timing to prompt engagement,

RESULTING IN better outcomes than mismatched timing (e.g. 'not the right time').

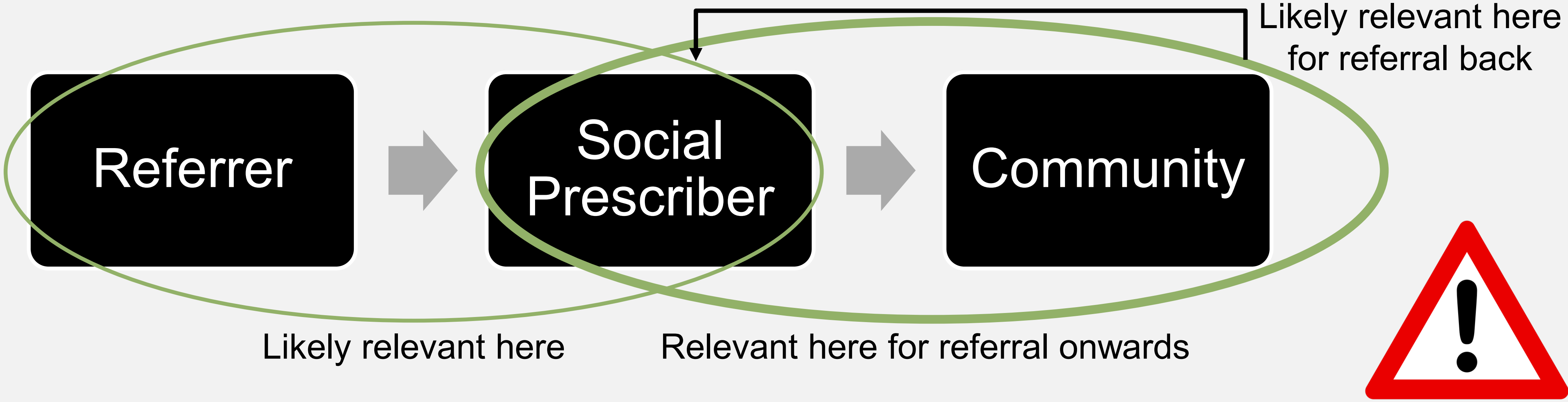

UNIVERSITÄT BIELEFELD

GRASP Uncertainties

A5. Mainstream Service Integration

Very weak

IF refugee-specific interventions function as bridges to mainstream services (building capacity, cultural navigation, warm handoffs),

THEN refugees develop sustainable access beyond intervention period,

RESULTING IN long-term integration;

IF time-limited interventions create parallel systems without mainstream service connections,

THEN time-limited dependency occurs without integration pathways.

UNIVERSITÄT BIELEFELD

GRASP Uncertainties

3. We therefore infer that:

IF refugee-serving community organisations act as empowering bridges for refugees and asylum seekers to access SP or mainstream health services,

THEN they benefit from this facilitation that supports their access,

RESULTING IN greater long-term service integration and engagement.

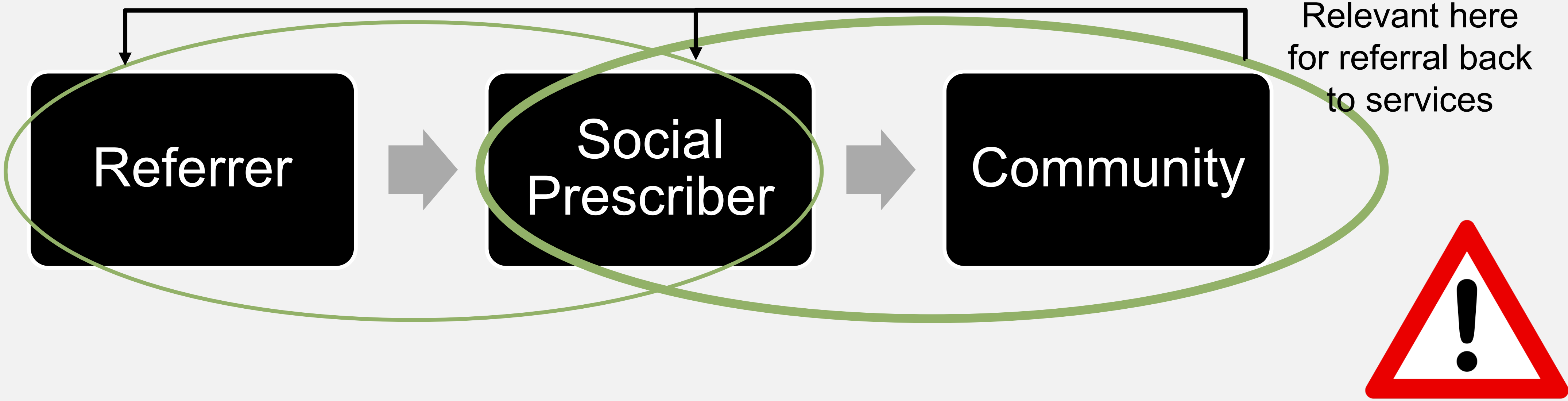

UNIVERSITÄT BIELEFELD

GRASP Uncertainties

A6. Failure Conditions and Contraindications

Weak

IF populations are in acute crisis (homelessness, extreme poverty, imminent deportation, acute mental health crisis) OR organizations lack essential infrastructure for chosen family type,

THEN even well-designed interventions fail as survival imperatives override engagement or implementation cannot proceed,

RESULTING IN non-participation or program collapse; contraindications include Family 4 without trusted location.

4. We therefore infer that:

IF refugees and asylum seekers are in acute crisis (e.g. homelessness, extreme poverty, imminent deportation or removal, change in status, acute mental health crisis),

THEN even well-designed non-crisis services or community organisations may not be appropriate for them,

RESULTING IN likely risk of non-participation or non-attendance.

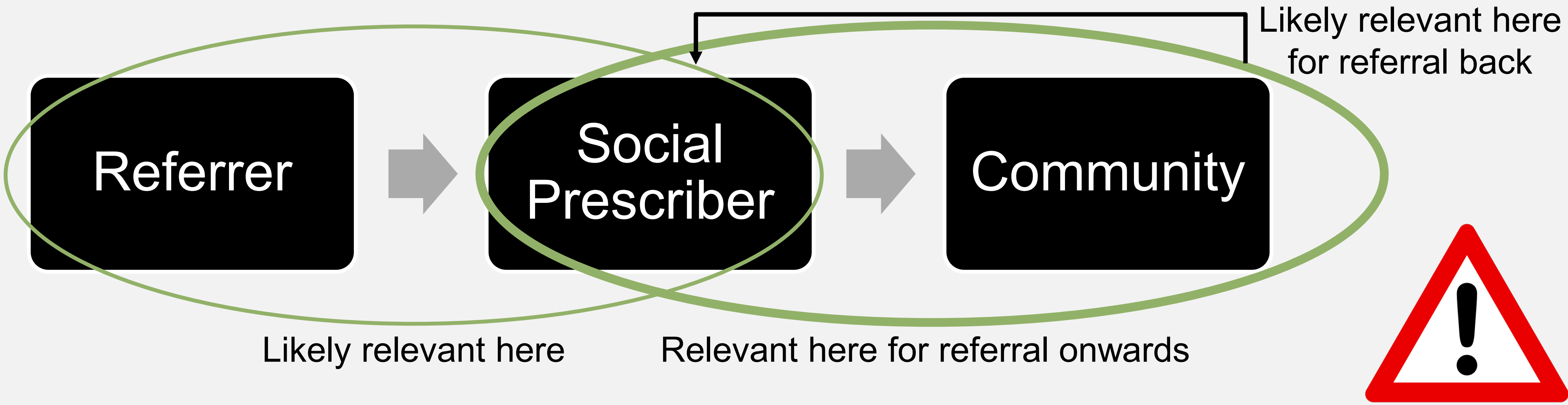

UNIVERSITÄT BIELEFELD

GRASP Uncertainties

A7. Intersectional Marginalization Response

Very weak

IF interventions are responsive to multiply-marginalized refugee individuals (refugees with disabilities, LGBTQ+ refugees, elderly, unaccompanied minors) with specialized adaptations beyond standard enablers,

THEN intersectionally marginalized refugees can access and benefit,

RESULTING IN within-group equity;

IF interventions assume refugee homogeneity,

THEN most marginalized remain excluded from refugee-specific services.

UNIVERSITÄT BIELEFELD

GRASP Uncertainties

5. We therefore infer that:

IF GP services, social prescribing services, and community services are ready for and responsive to the specific needs of multiply-marginalized refugees and asylum seekers,

THEN these individuals are more likely to access and benefit,

RESULTING IN greater equity of access and quality of care.

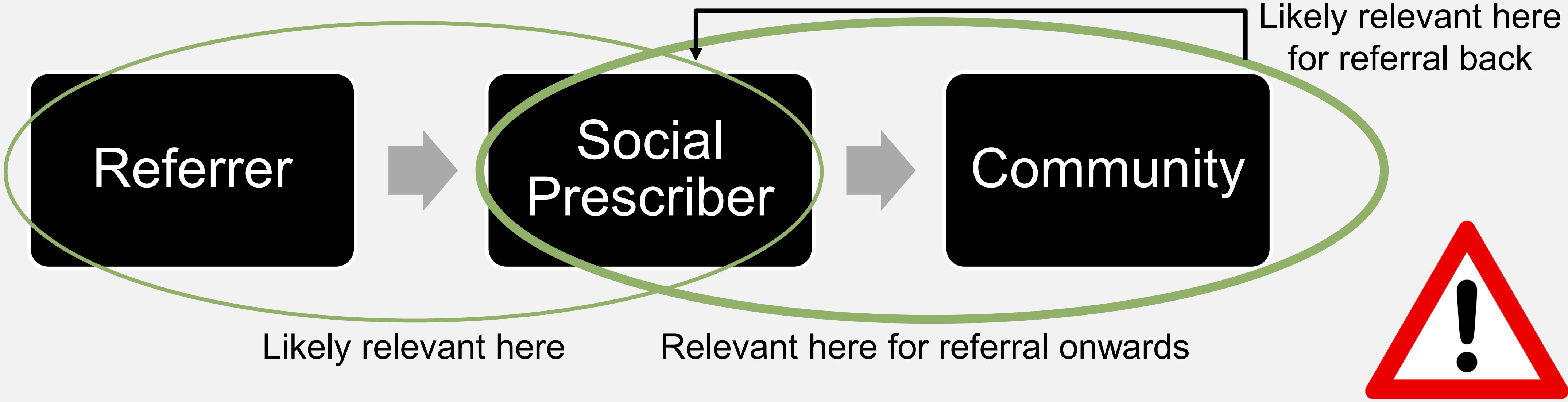

In Social Prescribing Appointments

UNIVERSITÄT BIELEFELD

GRASP Uncertainties

S4. Co-Production Domain Specificity

Moderate

IF interventions focus on navigation (Family 2) or trauma (Family 3) where refugee lived experience provides unique expertise,

THEN co-production/peer leadership activates cultural brokerage and shared witness mechanisms,

RESULTING IN enhanced effectiveness; professional facilitation may suffice for other intervention types.

6. We therefore infer that:

IF SP appointments involve navigation of services and/or trauma disclosure with refugee and asylum seekers,

THEN co-production of a wellbeing plan and ‘peer’ connection help recognize refugee lived expertise, bridge knowledge gaps, and individuals feeling ‘seen’ and ‘heard’,

RESULTING IN more effective support, meaning that SPs’ empathy, refugee-specific insight and potentially their shared personal characteristics matter to outcomes.

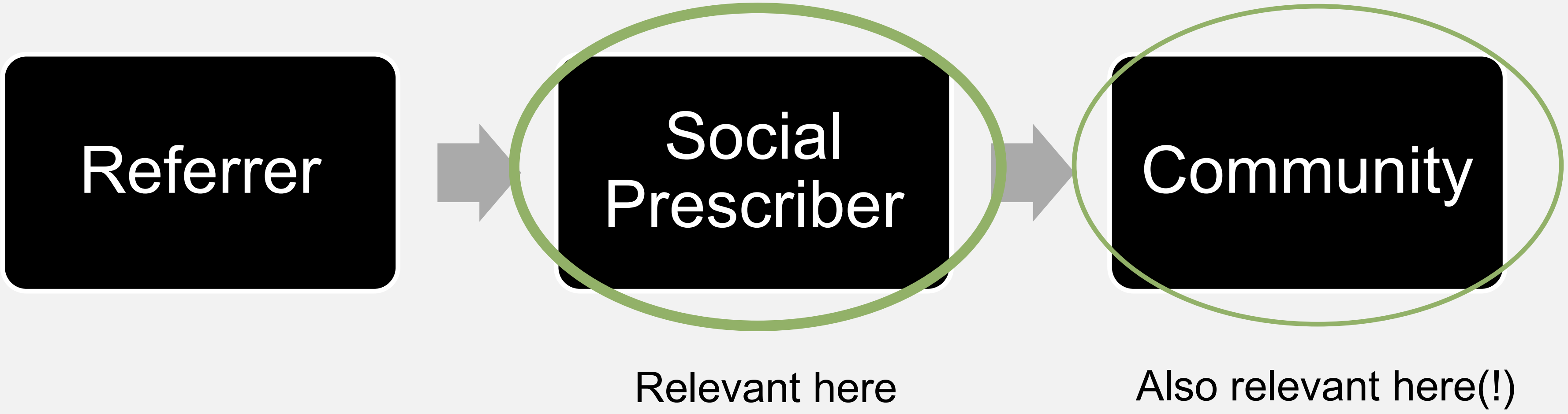

F2. Navigation Requires Refugee Experiential Expertise

Strong

IF system navigation interventions are co-produced with refugees,

THEN cultural brokerage mechanisms activate through lived experience knowledge of barriers and culturally-calibrated strategies,

RESULTING IN effective navigation of refugee-specific barriers that professionals cannot anticipate.

7. We therefore infer that:

IF SP appointments focusing on navigation of services with refugees and asylum seekers are co-produced in a way where SPs actively create space for their input,

THEN they can share their culturally specific needs and suggest strategies that genuinely fit their context,

RESULTING IN greater chance of navigating barriers that SPs didn't know about.

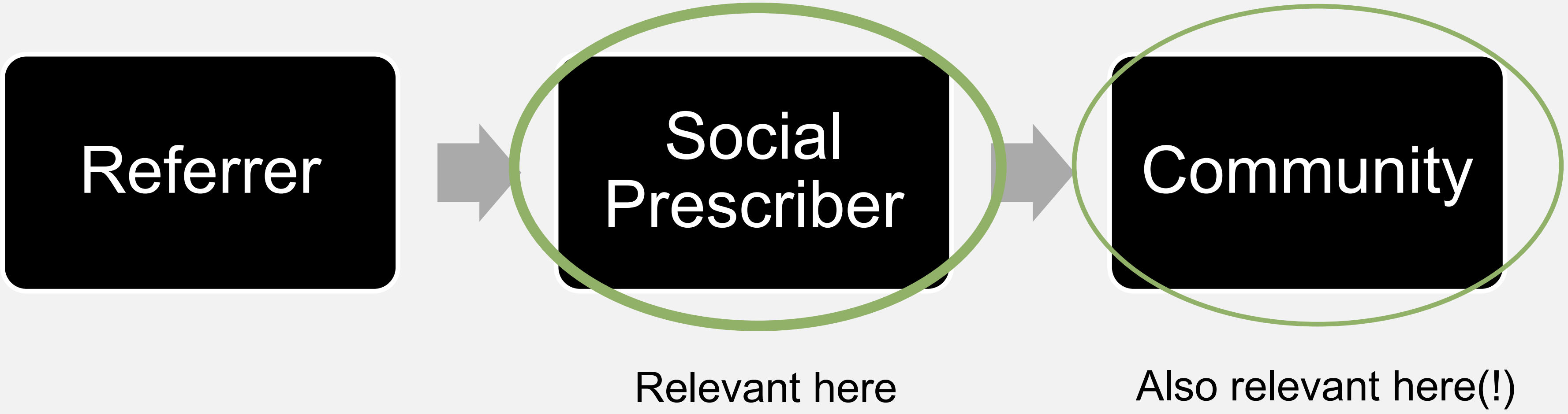

A3. Peer Leadership Scope and Support

Moderate

IF peer leaders are provided with training, supervision, compensation, and clearly defined roles (navigation, trauma support, health education),

THEN they activate cultural brokerage and shared witness mechanisms while maintaining wellbeing,

RESULTING IN sustained effective peer leadership; without support infrastructure, burnout and role confusion undermine effectiveness.

8. We therefore infer that:

IF SPs receive training, supervision, and compensation, with clearly defined roles (e.g. addressing navigation of services, trauma disclosure, health literacy)

THEN they can draw on their skills and experience while reducing risk of burnout and role confusion,

RESULTING IN more sustainable effective roles in SP.

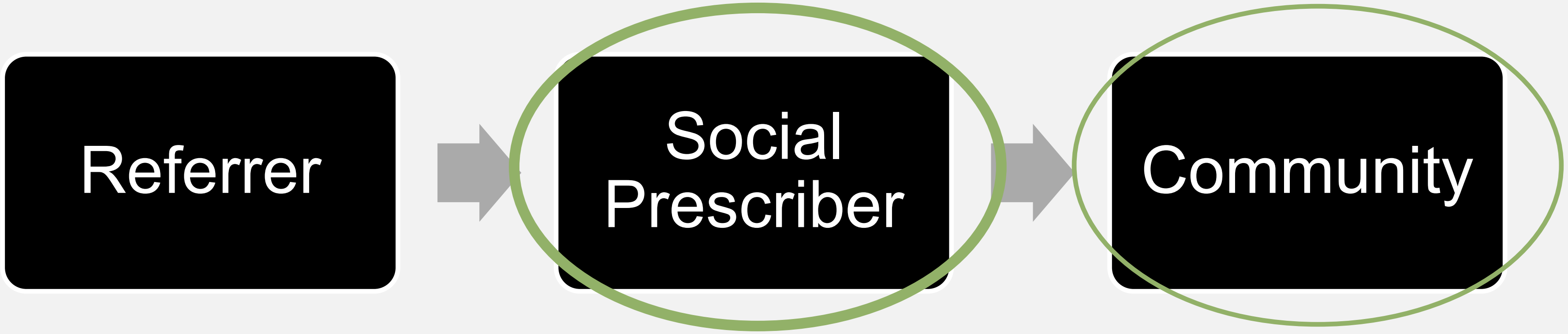

Relevant here

Also relevant here(!)

S3. Resource-Type Prioritization

Moderate

IF interventions strategically prioritize resource types matching their goals (material for barrier reduction, knowledge for navigation, relational for trauma, structural for social capital),

THEN moderate enabler provision (2-4) achieves effectiveness through targeted allocation,

RESULTING IN positive outcomes without comprehensive material support.

9. We therefore infer that:

IF SPs can identify and prioritize the right resources for the needs and risks in the appointment (e.g. service knowledge for navigation of services or relational connection for trauma disclosure),

THEN relevant further supports need to be in place (e.g. interpretation, referrals onwards)

RESULTING IN positive outcomes.

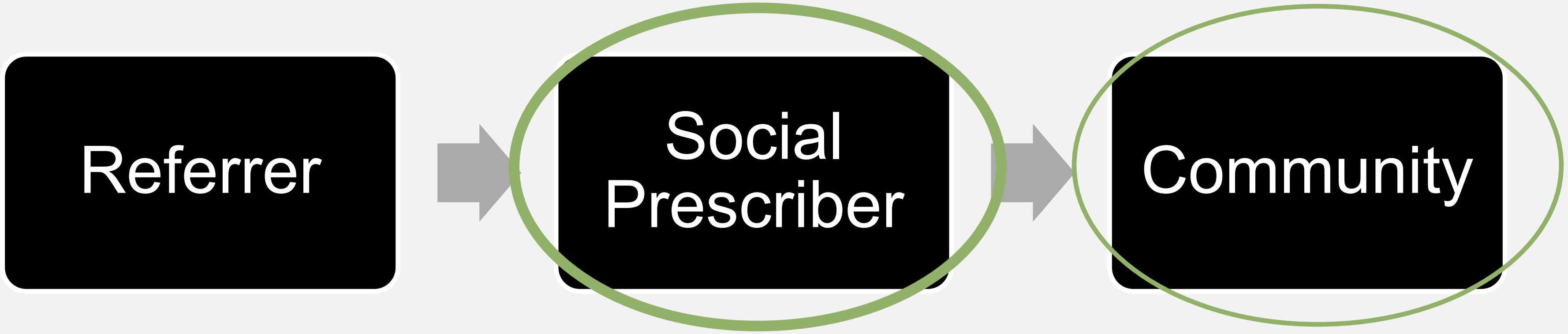

Relevant here

Also relevant here(!)

Onward referral from Social Prescribing

UNIVERSITÄT BIELEFELD

GRASP Uncertainties

S1. Burden-Intensity Matching

Moderate - Strong

IF refugees face multiple intersecting barriers (e.g. recent arrival, language skills, childcare needs, acute trauma),

THEN they require comprehensive enabler provision (>4 types, e.g. food, transport, childcare, interpretation, equipment, community-led staff etc.) to take up a referral to access services or participate in community activities,

RESULTING IN higher likelihood of attendance vs. risk of non-attendance when referred onward to minimal-support models.

10. We therefore infer that:

IF SPs are supporting refugees and asylum seekers experiencing multiple barriers who would like to be referred onwards to community organisations,

THEN referrals can be made to community organisations with the capacity to meet those needs through addressing specific barriers (e.g. interpretation, childcare, trusted location, transport),

RESULTING IN them being more likely to attend and engage.

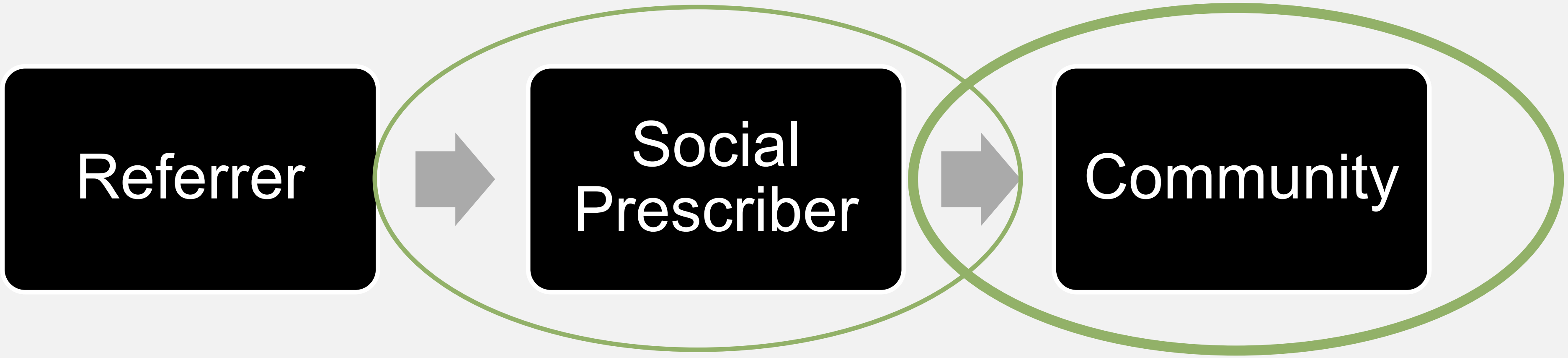

Likely relevant here

Relevant here for referral onwards

F3. Trauma Work Requires Safety Architecture + Peer Connection

Strong

IF trauma interventions include trauma-informed approaches, peer leadership, and cultural adaptation,

THEN safety architecture and shared witness mechanisms activate,

RESULTING IN trauma processing and recovery with moderate enabler provision (2-4 types).

UNIVERSITÄT BIELEFELD

GRASP Uncertainties

11. We therefore infer that:

IF SPs can connect refugees and asylum seekers with community organisations that use trauma-informed approaches, peer leadership, and are culturally appropriate,

THEN these can create safer spaces where participants feel heard and validated with the right further support (e.g. childcare, interpretation, trusted location, community-led staff),

RESULTING IN the potential to support in trauma processing and recovery.

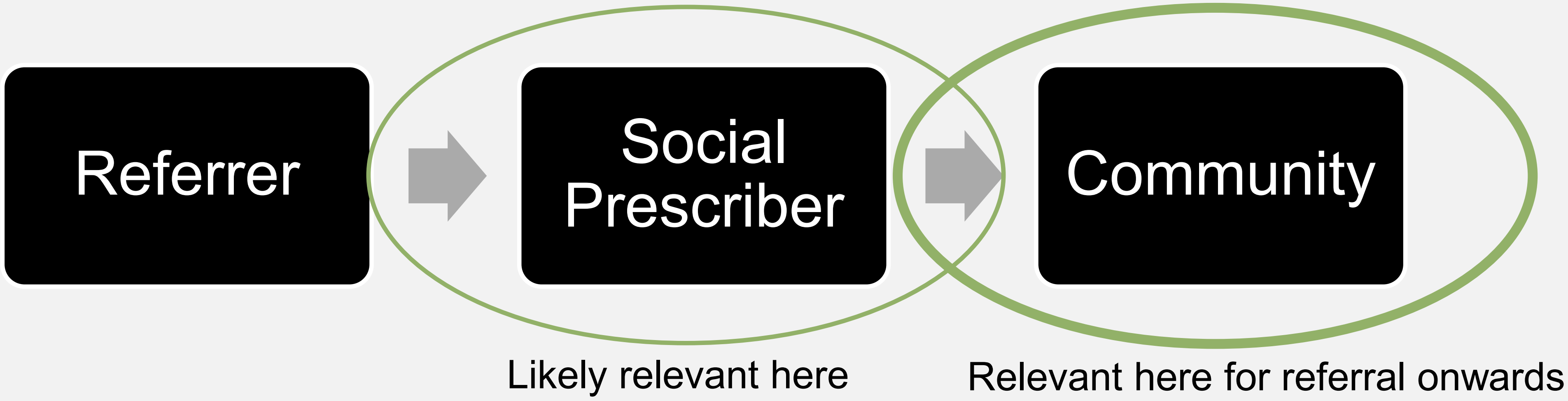

F4. Social Capital Benefits From Trusted Location And Exchange Infrastructure

Strong

IF social capital interventions provide trusted locations and opportunities for social exchange where refugees feel safe forming relationships,

THEN spatial safety mechanisms legitimize connection beyond survival networks,

RESULTING IN bridging social capital formation; without trusted locations, social risk-taking for new relationships cannot occur.

UNIVERSITÄT BIELEFELD

GRASP Uncertainties

12. We therefore infer that:

IF SPs can connect refugees and asylum seekers to community organisations that provide trusted locations and social exchange where they feel safe,

THEN the sense of physical and psychological safety in these social surroundings makes new connections possible,

RESULTING IN increased openness, creating the potential for forming positive new relationships.

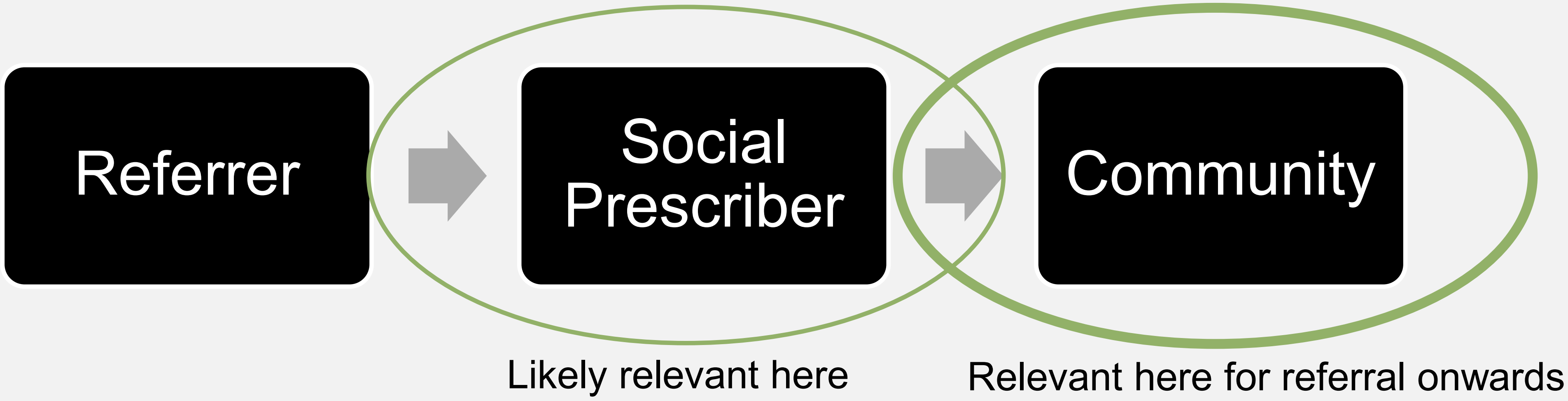

F5. Skills Transfer Assumes Capacity

Moderate

IF skills interventions provide minimal enablers (1-3) for established refugees with independent access capacity,

THEN efficient skills transfer occurs,

RESULTING IN cost-effective scalability;

IF highly marginalized refugees are directed to these models,

THEN unaddressed barriers prevent access,

RESULTING IN systematic exclusion regardless of content quality.

13. We therefore infer that:

IF SPs can connect settled refugees and asylum seekers who have existing agency and capacity with training programmes which include minimal further support or accompaniment,

THEN these individuals can benefit through learning or improving skills,

RESULTING IN scalable and cost-effective skills transfer programmes.

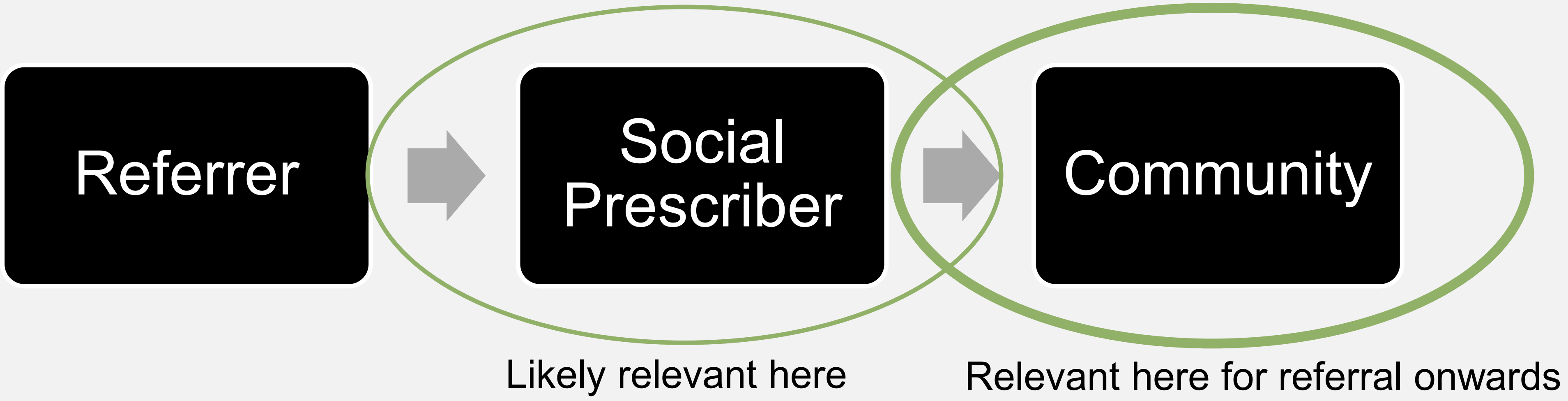

C3. Organizational Capacity Requirements

Moderate

IF organizations possess capacity matching intervention family needs (Family 1: funding/logistics; Family 2: co-production infrastructure; Family 3: trauma-informed culture; Family 4: trusted spaces; Family 5: minimal infrastructure),

THEN interventions can be implemented as designed,

RESULTING IN intended outcomes; capacity gaps undermine implementation regardless of design quality.

UNIVERSITÄT BIELEFELD

GRASP Uncertainties

14. We therefore infer that:

IF community organisations receiving social prescribing referrals have the capacities and resources they require (e.g., funding/logistics; co-production infrastructure; trauma-informed culture; trusted spaces)

THEN they can deliver activities or services as intended,

RESULTING IN achieving outcomes.

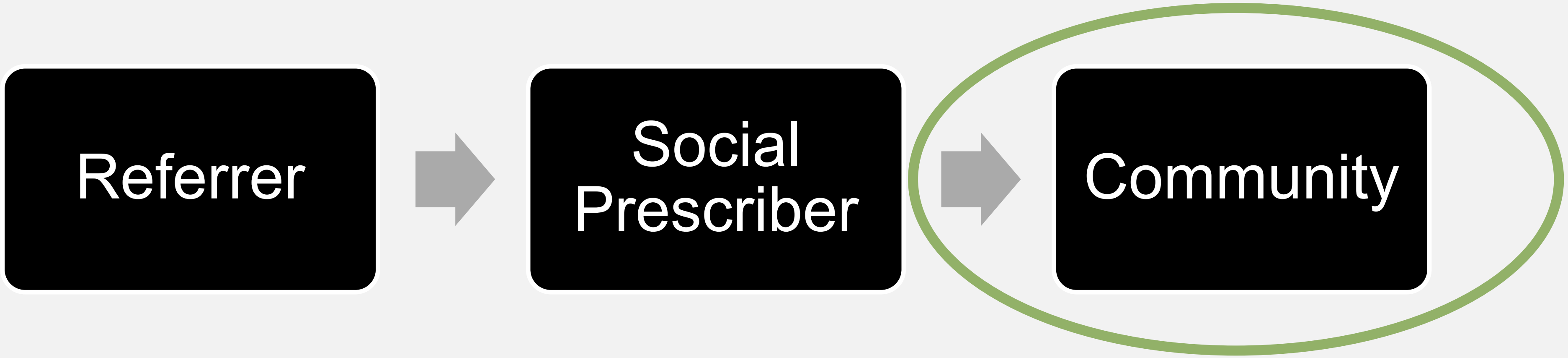

Relevant here for referral onwards

C4. Gender and Family Structure Considerations

Strong

IF interventions target women with children,

THEN childcare provision activates participation mechanisms by removing caregiving barriers,

RESULTING IN mothers' attendance and engagement; family-oriented interventions require different enablers than individual-focused.

UNIVERSITÄT
BIELEFELD

GRASP
Uncertainties

15. We therefore infer that:

IF SPs engage with women with young children,

THEN connecting them with community organisations that provide childcare or spaces where children are welcome removes caregiving barriers,

RESULTING IN greater likelihood of mothers' attendance and engagement.

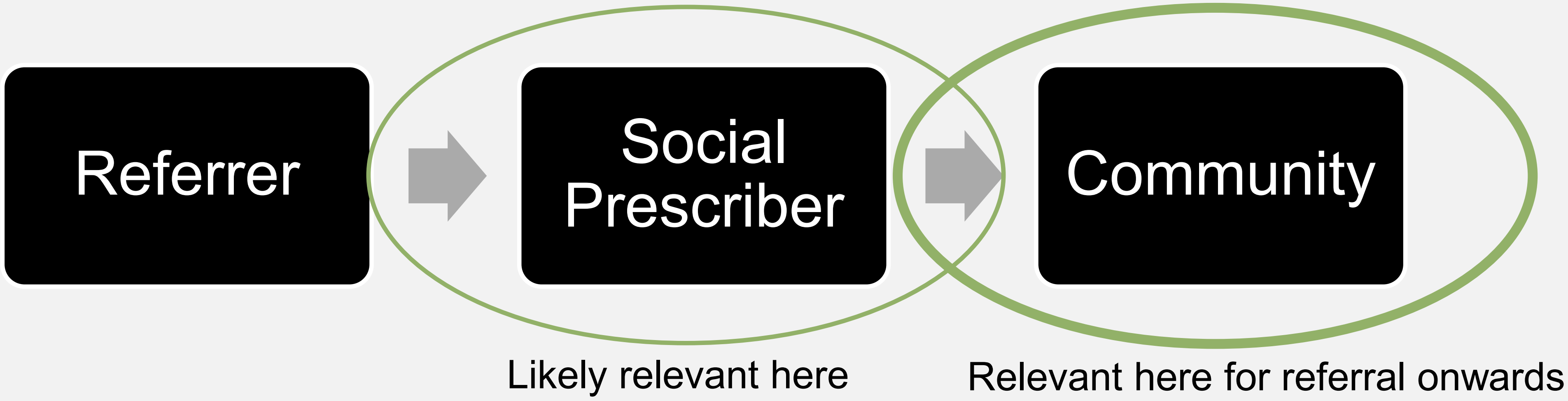

What happens next

- I complete my conversations with experts
- I write up these results, working in your feedback and prioritisation of statements
- This gets submitted in December 2025
- You can be acknowledged for your input -> **Do you want that?**

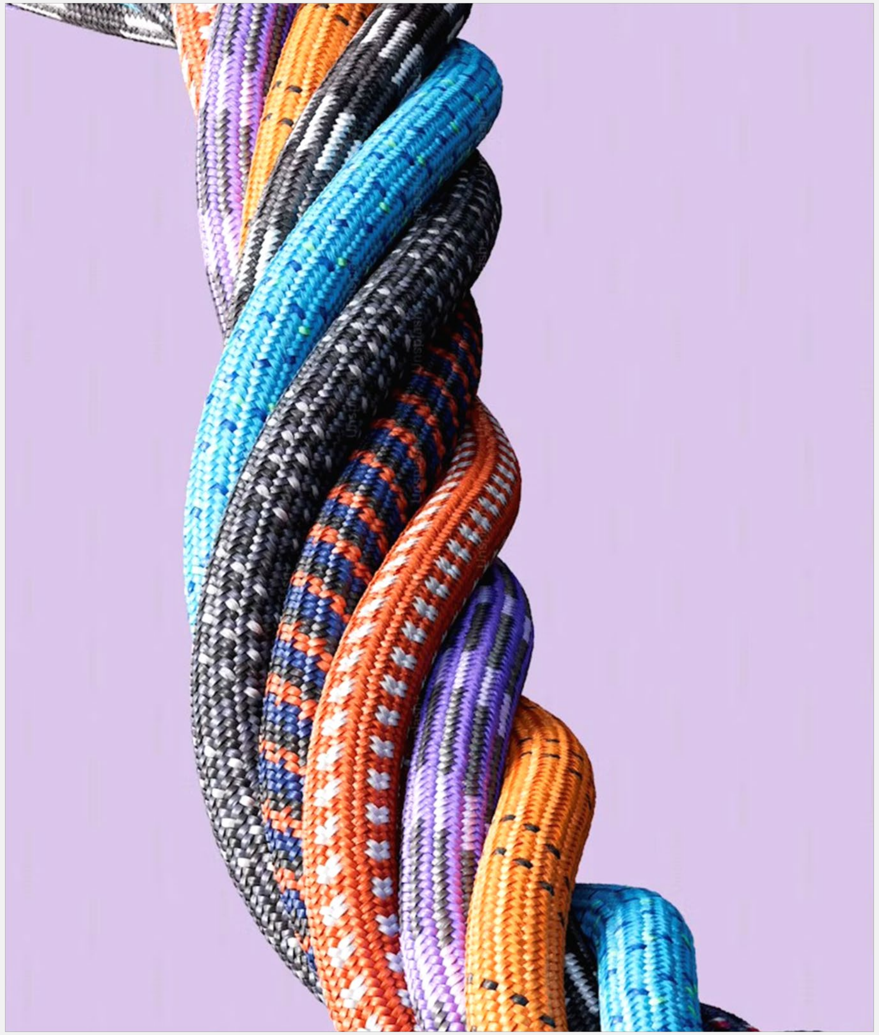

Final thoughts for today

Is there anything you wanted to share that you haven't done yet?

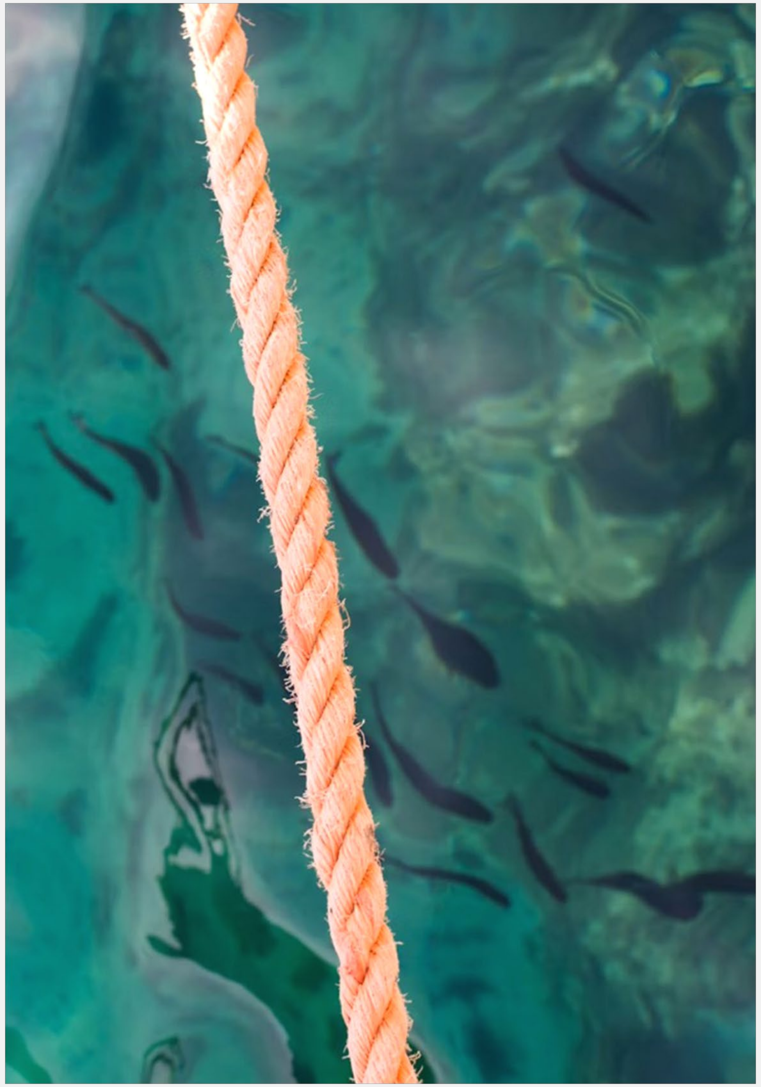

UNIVERSITÄT BIELEFELD

GRASP Uncertainties

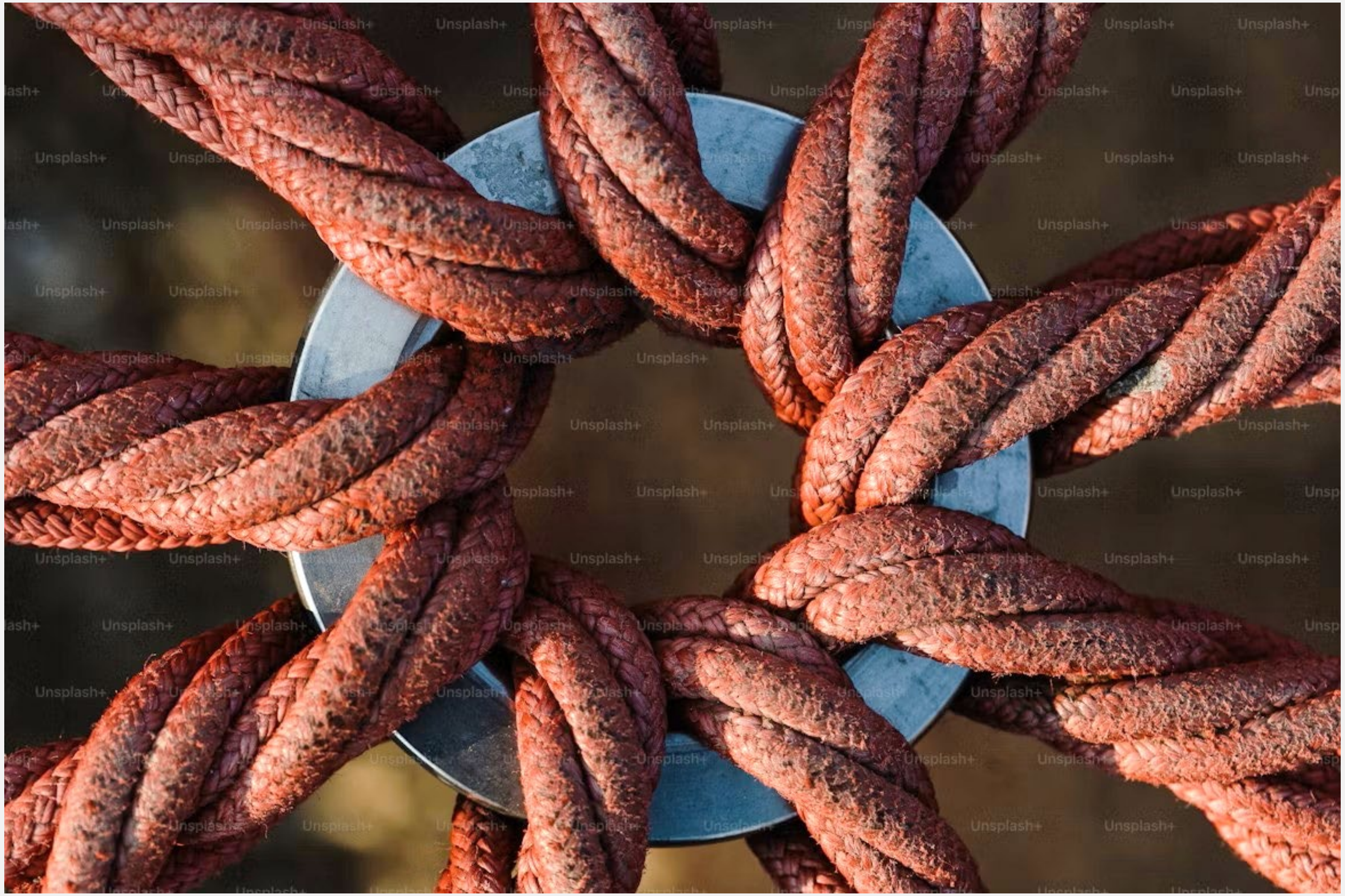

Thank you for participating!
